# Effects of non-digestible carbohydrates on gut microbiota and microbial metabolites: a randomised, controlled dietary intervention in healthy individuals

**DOI:** 10.1017/S000711452400271X

**Published:** 2024-11-04

**Authors:** Fiona C. Malcomson, Panayiotis Louca, Andrew Nelson, Naomi D. Willis, Iain McCallum, Long Xie, Arthur C. Ouwehand, Julian D. Stowell, Tom Preston, Douglas J. Morrison, Seamus B. Kelly, D. Michael Bradburn, Nigel J. Belshaw, Ian T. Johnson, Bernard M. Corfe, Christopher J. Stewart, John C. Mathers

**Affiliations:** 1 Human Nutrition and Exercise Research Centre, Centre for Healthier Lives, Population Health Sciences Institute, Newcastle University, Newcastle upon Tyne, NE2 4HH, UK; 2 Centre for Cancer, Population Health Sciences Institute, Newcastle University, Newcastle upon Tyne, UK; 3 Department of Applied Science, Northumbria University, Newcastle upon Tyne, NE1 8ST, UK; 4 Northumbria Healthcare NHS Foundation Trust, North Tyneside General Hospital, Rake Lane, North Shields, NE29 8NH, UK; 5 International Flavors & Fragrances, 10210 Kantvik, Finland; 6 DuPont Nutrition & Biosciences, Reigate, UK; 7 Scottish Universities Environmental Research Centre, College of Science and Engineering, University of Glasgow, Glasgow, UK; 8 Northumbria Healthcare National Health Service Foundation Trust, Ashington, UK; 9 University of East Anglia, Norwich Research Park, Norwich, NR4 7TJ, UK; 10 Quadram Institute, Norwich Research Park, Norwich, Norfolk, NR4 7UQ, UK; 11 Translational and Clinical Research Institute, Newcastle University, Newcastle upon Tyne, NE2 4HH, UK

**Keywords:** Non-digestible carbohydrates, resistant starch, polydextrose, gut microbiota, short-chain fatty acids, randomised controlled trial, dietary intervention, humans

## Abstract

The gut microbiome is impacted by certain types of dietary fibre. However, the type, duration, and dose needed to elicit gut microbial changes, and whether these changes also influence microbial metabolites, remains unclear. This study investigated the effects of supplementing healthy participants with two types of non-digestible carbohydrates (resistant starch (RS) and polydextrose (PD)), on the stool microbiota and microbial metabolite concentrations in plasma, stool, and urine, as secondary outcomes in the Dietary Intervention Stem Cells and Colorectal Cancer (DISC) Study. The DISC Study was a double-blind, randomised controlled trial that supplemented healthy participants with RS and/or PD or placebo for 50 days in a 2*2 factorial design. DNA was extracted from stool samples collected pre- and post-intervention, and V4 16S rRNA gene sequencing was used to profile the gut microbiota. Metabolite concentrations were measured in stool, plasma, and urine by high-performance liquid chromatography.

A total of 58 participants with paired samples available were included. After 50 days, no effects of RS or PD were detected on composition of the gut microbiota diversity (alpha- and beta-diversity), on genus relative abundance, or on metabolite concentrations. However, Drichlet’s multinomial mixture clustering-based approach suggests that some participants changed microbial enterotype post-intervention.

The gut microbiome and faecal, plasma, and urinary microbial metabolites were stable in response to a 50-day fibre intervention in middle aged adults. Larger and longer studies, including those which explore the effects of specific fibre sub-types, may be required to determine the relationships between fibre intake, the gut microbiome, and host-health.

## List of abbreviations

ATIMA: Agile Toolkit for Incisive Microbial Analysis
BCFA: branched-chain fatty acids
BMI: body mass index
CRC: colorectal cancer
DISC: Dietary Intervention Stem Cells and Colorectal Cancer
DMM: Dirichlet’s multinomial mixture
GLM: General linear model
LSM: least square mean
MTBE: methyl tert-butyl ether
OTUs: operational taxonomic units
PD: polydextrose
RCT: randomised controlled trial
RS: resistant starch
SCFA: short-chain fatty acids
SD: standard deviation
SMD: standard mean difference
SUERC: Scottish Universities Environmental Research Centre
VFA: volatile fatty acids

## Introduction

Dietary fibre has been defined as carbohydrate polymers with three or more monomeric units that are not hydrolysed by endogenous enzymes in the small intestine^(1)^. It reaches the large intestine and is fermented by resident gut bacteria to produce microbial metabolites such as short-chain fatty acids (SCFA) including acetate, propionate, and butyrate. Observational data suggest that higher intakes of dietary fibre, when compared with the lowest consumers, are associated with reduced risk of non-communicable diseases, including colorectal cancer, diabetes, and cardiovascular disease^(2)^. The proposed mechanisms through which dietary fibres exert their beneficial health effects include modulation of the gut microbiota and synthesis of microbial metabolites, primarily SCFAs.

Dietary fibres include a wide range of non-digestible carbohydrates that differ in terms of physiochemical properties such as solubility, and physiological effects such as fermentability^(1)^. These non-digestible carbohydrates include resistant starch (RS; the sum of starch and products of starch digestion that are not absorbed in the small bowel^(3)^) and polydextrose (PD; a synthetic, soluble fibre and glucose polymer with sorbitol end groups developed as a low-calorie sweetener with bulking properties^(4)^). Further, five subtypes of RS exist according to the factors affecting their resistance to digestion in the colon: type 1 (physically inaccessible e.g., wholegrains), type 2 (ungelatinised resistant granules with type B crystallinity e.g., high-amylose maize starch, green bananas), type 3 (retrograded starch e.g., cooked then cooled potatoes), type 4 (chemically modified starches e.g., cross-linked starch in thickeners), and type 5 (amylose-lipid complexes e.g., palmitic acid amylose complex, foods with high amylose content)^(5)^. Some highly fermentable fibres, including fructooligosaccharides and inulin, exert prebiotic effects, i.e., selectively stimulate the growth or activity of one or a limited number of gut bacteria, and thus confer beneficial effects on host health^(6)^. A recent systematic review and meta-analysis, spanning 64 studies and over 2,000 participants, investigated the effects of dietary fibre interventions on gut microbiota composition in healthy adults and included eight randomised controlled studies which supplemented with RS and/or PD^(7)^. In the meta-analysis, six studies reported Shannon diversity index and three reported total number of observed operational taxonomic units (OTUs) and showed no overall effect of dietary fibre supplementation on alpha-diversity compared with placebo or low-fibre comparators^(7)^. Subgroup analysis of the effects of fibres classified as candidate prebiotics (which include RS and PD) on bacterial abundance demonstrated increased *Bifidobacterium* spp., but no effects on *Lactobacillus* spp. (the two most commonly reported taxa), compared with placebo or low-fibre controls^(7)^. It is not only the type of dietary fibre that is important, but fibre subtypes (e.g., RS type 1 vs. type 2) may have differential effects on the gut microbiota and on host health. The authors also explored effects of dietary fibre interventions (but did not perform fibre type subgroup analyses) and reported an increase in faecal butyrate concentrations after dietary fibre interventions compared with placebo or low-fibre comparators (standard mean difference (SMD) [95%CI]: 0.24 [0.00, 0.47], *p* = 0.05)^(7)^. However, no effects on total faecal SCFA concentrations were observed^(7)^.

The SCFA butyrate exerts chemoprotective and other health-promoting properties that may be beneficial in the prevention or management of diseases such as colorectal cancer, insulin resistance, and hypercholesterolemia^(8)^. Chambers et al. have also shown that targeted delivery of propionate has positive effects on appetite regulation, body weight maintenance, and adiposity, and modulated the gut microbiota and plasma metabolome^(9; 10)^. In contrast, higher intakes of dietary fibre, particularly insoluble fibre, have been associated with lower concentrations of faecal branched-chain fatty acids (BCFA)^(11)^, endproducts of protein decomposition, which may provide an additional chemoprotective effect as certain metabolites produced during this process are carcinogenic. For example, in healthy male volunteers, supplementation with PD or soluble maize fibre for 21 days reduced the production of putrefactive compounds, including faecal BCFAs and amomonia^(12)^.

The aim of this study was to investigate the effects of supplementing healthy participants for 50 days with two types of non-digestible carbohydrates which fall within the definition of dietary fibre (RS: Hi-maize® 260 (Ingredion™, type 2 RS), and/or PD: Litesse® *Ultra*™ (International Flavors & Fragances™ Danisco®)) on the gut microbiota and on microbial metabolite concentrations, including SCFAs and BCFAs, in stool, plasma, and urine in the Dietary Intervention Stem Cells and Colorectal Cancer (DISC) Study. This is a secondary analysis of samples and data from the DISC Study. We have reported previously the effects of RS and PD on a range of outcomes including inflammatory markers, WNT pathway-related markers, colorectal crypt cell proliferative state, and microRNA expression^(13; 14; 15)^.

## Experimental Methods

**Figure 1:**
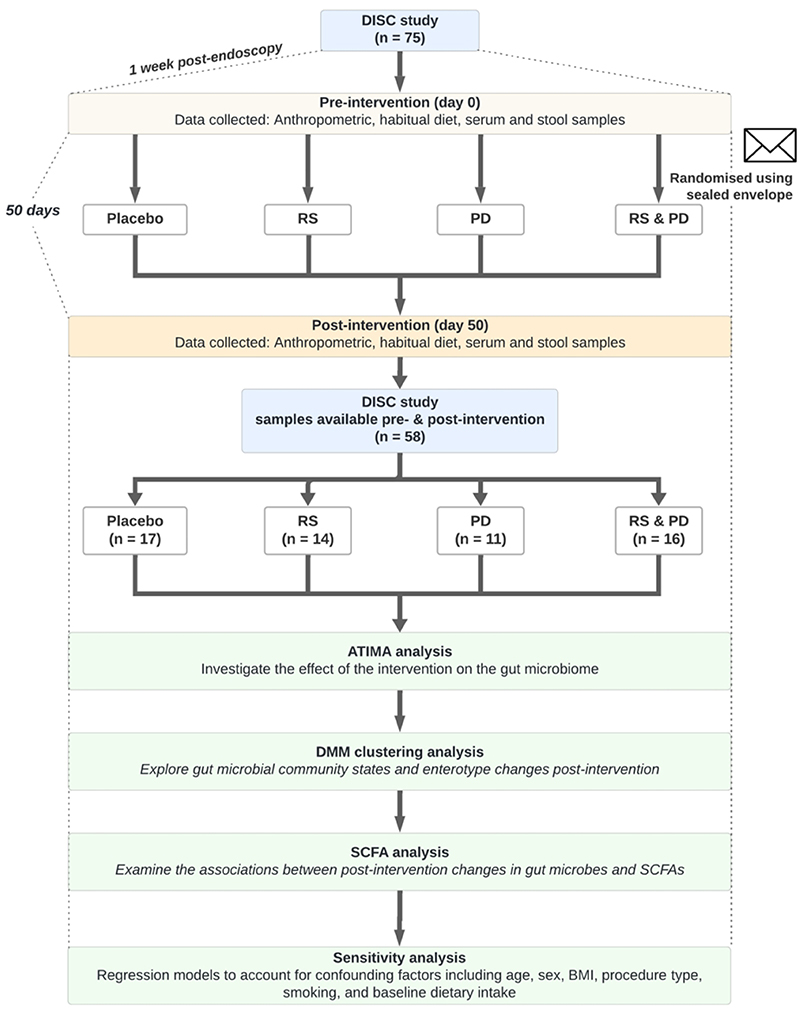
Flowchart of DISC study design and analytical pipeline. Abbreviations: DISC, Dietary Intervention Stem Cells and Colorectal Cancer; ATIMA, Agile Toolkit for Incisive Microbial Analysis; BMI, body mass index; DMM, Dirichlet’s multinomial mixture; PD, polydextrose; RS, resistant starch; SCFA, short-chain fatty acids.

A flowchart of the study design and analytical pipeline is presented in Figure 1.

### The DISC Study

The DISC Study was a double-blind, randomised, placebo controlled dietary intervention that supplemented 75 healthy participants with two types of non-digestible carbohydrates (RS and/or PD) or respective placebos in a 2*2 factorial design for 50 days^(13)^. Participants were recruited by the research team from gastroenterology outpatients departments at North Tyneside General Hospital and Wansbeck General Hospital in the North East of England between May 2010 and July 2011 using endoscopy patient lists. Potential participants were sent a study invitation letter with detailed information about the study at least five days prior to their hospital appointment. At endoscopy, potential study participants were screened for exclusion criteria, which included: aged <16 or >85 years, pregnant or planning to become pregnant, diabetes mellitus, familial adenomatous polyposis syndrome, Lynch syndrome, known colorectal tumour or prior CRC, prior colorectal resection, active colonic inflammation at endoscopy, iatrogenic perforation at endoscopy, incomplete left-sided examination, colorectal carcinoma discovered at endoscopy or histology, chemotherapy in the last six months, administering non-steroidal anti-inflammatories, anti-coagulants or immunosuppressive medication. Ethical approval for the DISC Study was granted by the Newcastle and North Tyneside Research Ethics Committee on 10th December 2009 (REC No. 09/H0907/77) and Caldicott approval for the storage of data was provided by the Northumbria NHS Foundation Trust (C1792). All participants provided informed written consent. The clinical trial is registered with ClinicalTrials.gov (Identifier NCT01214681).

Habitual diet was assessed at baseline using a food frequency questionnaire (FFQ) adapted from that used in the EPIC-Norfolk study (version 6, CAMB/PQ/6/1205)^(16)^. Participants were asked to consume their normal diet throughout the study. At least one week after their baseline endoscopy procedure, participants were randomised to one of four groups by selecting a sealed, opaque envelope:

- Double placebo (12 g Maltodextrin (RS placebo) + 23g Amioca starch (PD placebo))
- PD (12g Litesse® *Ultra*™ (International Flavors & Fragances™ Danisco®)
- RS (23g Hi-maize® 260, Ingredion™, Food Innovation)
- RS + PD (23g Hi-maize® 260, Ingredion™, Food Innovation + 12g Litesse® *Ultra*™, International Flavors & Fragances™ Danisco®)

The allocation codes were locked and participants and the research team were blinded.

The RS utilised in this study was Hi-maize® 260 (Ingredion™), a type 2 RS that is isolated from high-amylose corn hybrids and occurs in the natural granular form and is approximately 53% RS, with the remaining 40% comprising digestible starch. PD was given in the form of Litesse® *Ultra*™, a sugar-free soluble fibre developed as a sweetener. It is a glucose polymer produced from sorbitol, dextrose, and citric acid, which are derived naturally from corn. The intervention agents were supplied in a white powdered form in foil sachets packed into boxes each containing a week’s worth of sachets. Participants consumed 35g of intervention supplement per day divided into four sachets (two sachets of each intervention agent). Participants were asked to consume the powdered supplements by adding to cold food or liquids such as yoghurt, orange juice or water. Participants were asked to retain all their sachets, including those that were not consumed. To measure compliance, the number of consumed and not consumed sachets were counted for each participant.

### Sample collection

Participants provided rectal mucosal biopsies, blood, spot urine, stool, and buccal cell samples at baseline (day 0) and post-intervention (day 50). Participants who underwent colonoscopy (pre-intervention only) were fasted at the point of blood sample collection. Blood samples were collected in seven 4ml BD Vacutainer® K3EDTA tubes (Becton Dickinson, UK) and one 5ml BD Vacutainer® SST™ II Advance tube with gold hemogard closure (Becton Dickinson, UK). EDTA tubes were centrifuged for 5 minutes at 31,00g and 4°C and plasma extracted and stored at -80° C until analysis.

At the initial endoscopy appointment, participants were provided with equipment for the collection of urine and stool at home. Sample collection was performed prior to the first home visit, seven days post-endoscopy appointment, to minimise any effects of bowel preparation associated with the endoscopy procedures. Post-intervention samples were collected just prior to the participant’s repeat sigmoidoscopy. For each collection, participants were provided with a large sealable bucket pot, a disposable bedpan, two ice packs and a cool bag. Urine and stool samples were kept in cool bags containing ice packs until collected by a member of the research team (pre-intervention samples) or brought to the repeat study visit (post-intervention samples). Stool was processed within 3-18hr of defecation. Samples were divided into 5-6 aliquots and stored immediately at −80°C until analysis.

### Gut microbiota sequencing

Gut microbiota analysis was performed ∼10 years after data/sample collection. DNA was extracted from 250mg stool samples using the DNeasy® PowerLyzer® PowerSoil® kit (Qiagen, UK) and following the manufacturer’s instructions. Bacterial profiling of the variable region 4 (V4) of the 16S rRNA gene was carried out by NU-OMICS (Northumbria University) based on the Schloss wet-lab MiSeq SOP ^(17)^. Briefly, PCR was carried out using 1x Accuprime Pfx Supermix, 0.5 µM each primer and 1 µl of template DNA under the following conditions 95°C 2 minutes, 30 cycles 95°C 20s, 55°C 15s, 72°C 5 minutes with a final extension 72°C 10 minutes. One positive (Zymobiomics Microbial Mock community DNA standard) and one negative control sample were included in each 96 well plate and carried through to sequencing. PCR products were quantified using Quant-iT™ PicoGreen™ dsDNA Assay (Invitrogen) and each sample was normalised to 10nM and then each 96 well plate was pooled. Each pool was quantified using fragment size determined by BioAnalyzer (Agilent Technologies) and concentration by Qubit (Invitrogen). Pools were combined in equimolar amounts to create a single library then denatured using 0.2N NaOH for 5 minutes and diluted to a final concentration of 5 pM, supplemented with 25% PhiX and loaded onto a MiSeq V2 500 cycle cartridge. Fastq files were processed using Mothur (v1.48). Paired reads were merged and then filtered to remove contigs >275bp, homopolymer >8 and any ambiguous base. The high quality reads were dereplicated and aligned to the Silva reference alignment (v132). Chimeric sequences were removed using VSearch^(18)^ and taxonomy was assigned using the RDP database (v18) and non Bacterial sequences were removed.

### Quantification of microbial metabolite concentrations in stool, urine, and plasma

Microbial metabolite analyses were performed within 12-24 months after data/sample collection. The concentrations of microbial metabolites (SCFA, BCFA, volatile fatty acids, lactic acid) in stool were quantified by gas chromatography by International Flavors & Fragances^™^ Danisco^®^, Finland as described previously^(19)^. Briefly, 1ml of 20mm pivalic acid and 5ml of water were added to 1g of faecal sample, mixed thoroughly, and centrifuged at 5,000g for 5 minutes. 250µl of saturated oxalic acid solution were added to 500µl of the supernatant and the mixture was incubated at 48°C for 60 minutes, before centrifugation at 16,000 g for 5 minutes. The supernatant fraction was used for analysis, with pivalic acid as the internal standard.

Plasma and urinary SCFA and BCFA concentrations were measured by Scottish Universities Environmental Research Centre (SUERC), East Kilbride, UK as described by Morrison et al.^(20)^. SCFA and BCFA were extracted and derivatised as tBDMS (tert-butyl dimethyl silyl) esters prior to deuterium dilution analysis by GCMS. An alkaline internal standard mix containing 3 deuterated SCFA, 3 deuterated BCFA and 3-methyl valerate was added to each sample. Plasma samples (0.3 mL) were mixed and deproteinised using an ultracentrifuge device (Amicon Ultra 0.5 mL 30 kDa, Merck). Urine samples (1 mL) were spiked with an alkaline internal standard mix, but not ultrafiltered. Both sample types were dried by vacuum centrifuge. Blank tubes, deuterium enriched SCFA standards and unenriched SCFA standards were also dried. Dry samples and standards were acidified with dilute HCl. A volume of methyl tert-butyl ether (MTBE) was added, and mixed to extract the SCFA. A sub-sample of the upper MTBE phase was pipetted into a GC vial and a composite derivatisation reagent was added. The freshly mixed derivatisation reagent contained tBDMSIM (Merck Sigma, UK) in acetonitrile with a hexanoic acid spike to facilitate removal of an acetate reagent blank. The vials were capped. They were derivatised at 70oC for 60 minutes and cooled. Samples and standards were analysed by GCMS (Agilent MSD, UK) in selected monitoring mode. SCFA and BCFA concentrations were calculated by deuterium dilution analysis.

### Statistical analyses

The DISC Study was not subject to a formal power calculation, and a target of seventy-five participants - allowing for a 10% dropout rate, was set based on our previous study^(21)^.

Statistical analyses were performed using R version 4.3.1^(22)^, and the Agile Toolkit for Incisive Microbial Analysis (ATIMA) (https://atima.research.bcm.edu/)^(23)^ developed by the Centre for Metagenomics and Microbiome Research at the Baylor College of Medicine. The R-based software, ATIMA, is a stand-alone tool for analysing and visualising trends in alpha and beta diversity, and taxa abundance. The rarefaction depth was set to 13428, at which all negative controls and sequencing negatives were removed from the dataset. At this depth, 31% of reads were used (1,879,920 / 6,097,817 reads retained). Microbial abundances with <10% prevalence were removed. Using ATIMA, Mann-Whitney test was used to compare pre- vs. post-intervention data according to intervention agent (RS, PD, or respective placebos). Differences in beta-diversity (weighted Bray-Curtis distance) were assessed using PERMANOVA, including study ID as a nesting factor. We controlled for false discovery rate (FDR) using the Bejamini-Hochberg procedure^(24)^ and a FDR < 0.05 was considered significant.

To cluster gut microbial community states and distinguish inter-individual variations in gut microbiome response to the intervention we conducted Dirichlet’s multinomial mixture (DMM) modelling^(25)^. The Laplace approximation criterion was used to determine the optimal number of clusters.

The ANOVA general linear model (GLM) was used to test for effects of RS and PD, and for interactions between the two, on post-intervention SCFA concentrations, adjusting for pre-intervention measurement, age, sex, BMI, endoscopy procedure, and smoking status as covariates. Additionally, we calculated and investigated (using the same approaches as above) changes in microbial and SCFA concentrations. Changes in microbial abundance and SCFA concentrations were calculated as delta = post-intervention value – corresponding pre-intervention value. Delta abundances were quantile normalised prior to modelling^(26)^. Confounding variables included age, sex, BMI, procedure type (flexible sigmoidoscopy or colonoscopy), smoking status, and baseline dietary factors (intakes of energy, fibre, and alcohol). Spearman’s correlations were used to explore metabolite correlations, both pre- and post-intervention, across tissue types (stool, urine, and plasma). We also utilised the MaAsLin2 R package to conduct mixed effect modelling and explore the links between gut microbiome composition and intervention response. Within mixed effect modelling, participant ID was modelled as a random effect, intervention and additional covariates were modelled as fixed effects.

## Results

### Participant characteristics

Fifty-eight DISC Study participants, for whom paired stool samples (pre- and post-intervention) were available for analyses, were included in this study (Figure 1), and their characteristics are summarised in Table 1. All participants identified as being White British. The mean age of participants across all groups was 53 (SD 12) years. Just over half of the included participants were female (55%). Most participants (83%) were classed as having overweight or obesity based on their BMI. Over two thirds of participants underwent endoscopic examination by flexible sigmoidoscopy during the baseline (pre-intervention) study visit. Half of the participants were never smokers, 26% were former smokers and 24% were current smokers. The mean dietary fibre (assessed using the Englyst method ^(27)^) intake at baseline was 22.5 g/day (SD 10.9). At baseline, there were no significant differences between intervention groups in habitual intake of energy (0.987), dietary fibre (p=0.942) or protein (p=0.532).

### Bacterial profiles in stool at baseline from DISC Study participants

Pre-intervention, the bacterial profiles in stool from the DISC Study participants reflected those of healthy human adults, as reported by King and colleagues^(28)^; Firmicutes and Bacteroidetes were the most dominant phyla, followed by Proteobacteria, Actinobacteria, and Verrucomicrobia (data not shown). At the genus level, the most abundant genera were *Bacteroides, Faecalibacterium, Prevotella, Alistipes*, and *Roseburia*. There were no differences in the relative abundance of these bacteria between the four intervention groups at baseline. However, at the genus level, the relative abundance of *Streptococcus* was lower in participants randomised to the double placebo arm (*FDR<0.05*).

### Effects of resistant starch and polydextrose on gut microbiota diversity

**Figure 2:**
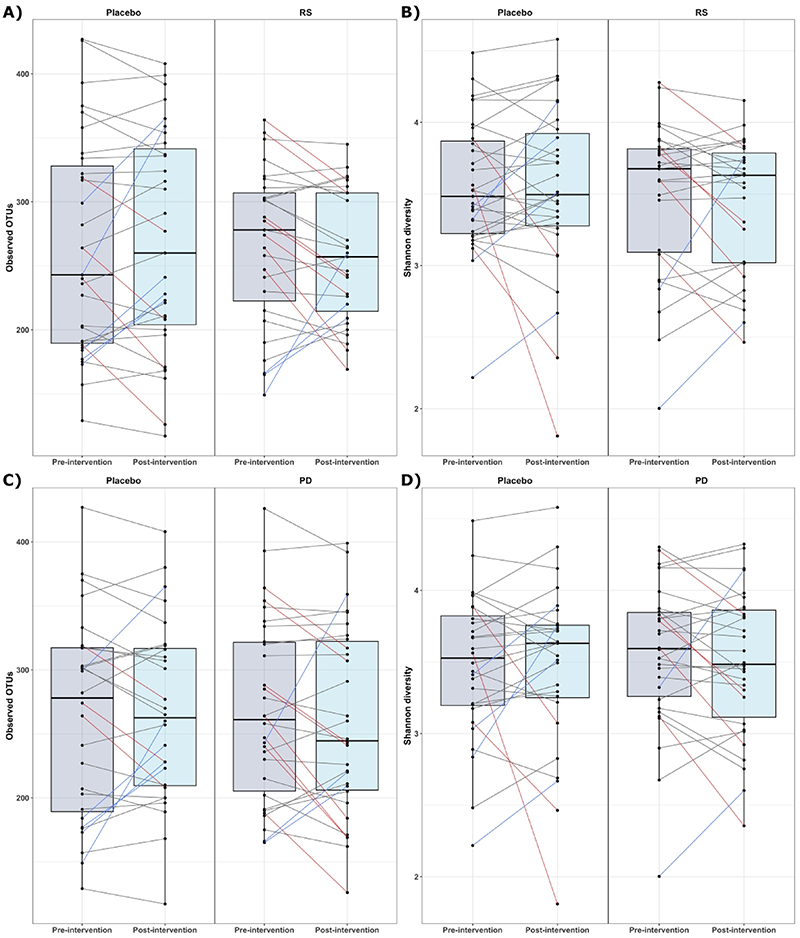
Effects of supplementation with RS and PD on two metrics of alpha diversity. Alpha diversity is represented by both observed operational taxonomic units (OTUs) and Shannon Diversity. Panels **A** and **B** show the impact of RS supplementation on alpha diversity measured by observed OTUs and Shannon diversity, respectively. Panels **C** and **D** illustrate the effects of PD supplementation on the same alpha diversity metrics. Each point represents individual participants pre- and post-intervention. Lines are coloured if change was > 1SD, red lines between paired points represent a decrease and blue lines an increase, black lines represent changes of < 1SD. *Abbreviations: RS, resistant starch; PD, polydextrose*.

When investigating effects of the intervention on alpha-diversity metrics (observed OTUs (richness) and Shannon index), no effects were detected post-intervention on alpha-diversity for either RS (observed OTUs, *p*=0.833; Shannon index, *p*=0.867) or PD (observed OTUs, *p*=0.77; Shannon index, *p*=0.89) (Figure 2).

**Figure 3:**
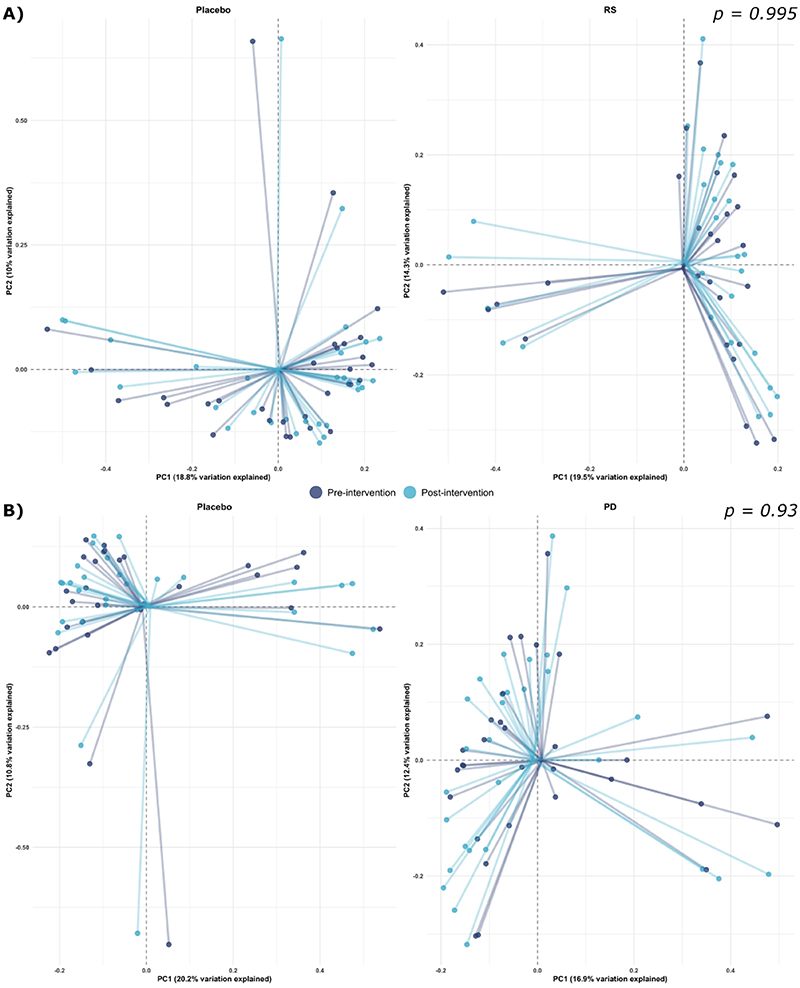
Principal Coordinates Analysis based on weighted Bray-Curtis distance metrics, illustrating microbial communities pre- and post-intervention in response to both interventions. **A)** Impact of RS supplement intervention on microbial community composition. **B)** Influence of PD supplement intervention on microbial community composition. *Abbreviations: RS, resistant starch; PD, polydextrose*.

We further tested the effect of the intervention on beta-diversity metrics using both weighted and unweighted Bray-Curtis distances and found no effect of supplementation with RS (weighted *p*=0.995, Figure 3; unweighted: *p*=1, Supplementary Figure 1) or PD (weighted: *p*=0.93, Figure 3; unweighted: *p=*0.998, Supplementary Figure 1) compared with placebo.

### Effects of resistant starch and polydextrose on gut microbiota abundance

**Figure 4:**
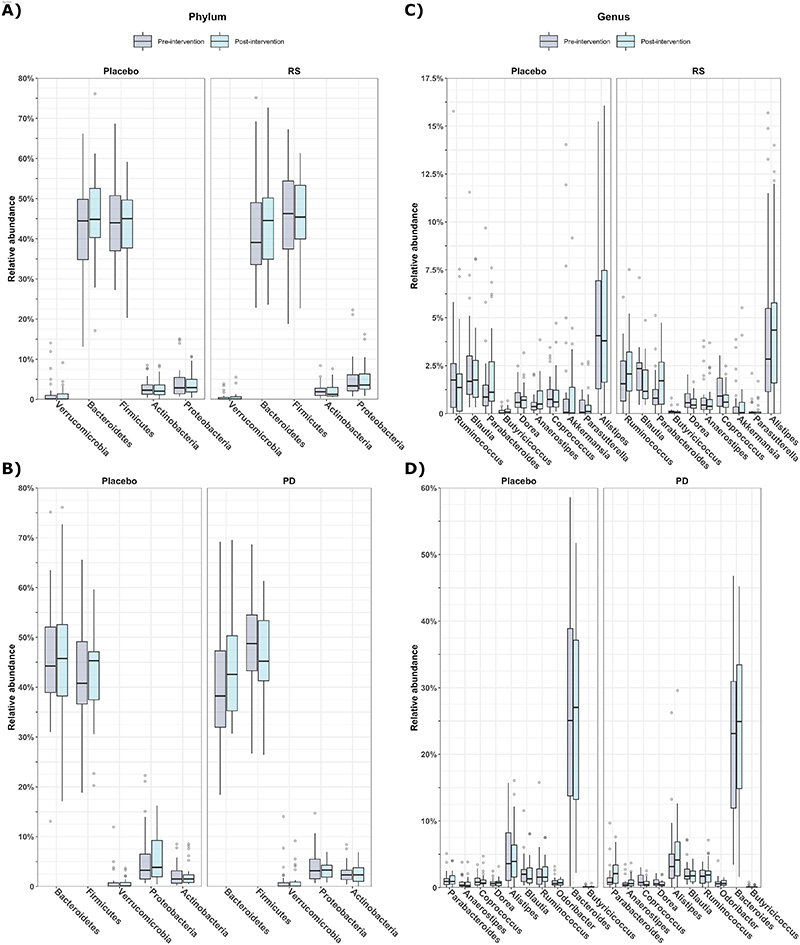
Effects of supplementation with A) RS and B) PD on the relative abundance of bacteria. Phyla and genera are ordered based on lowest p value. Boxes represent interquartile ranges, with lines denoting median. *Abbreviations: RS, resistant starch; PD, polydextrose*.

When investigating differences in relative abundance of bacteria at taxonomic levels, we detected no effects of either RS or PD compared with their respective placebos at the phylum or genus level (Figure 4).

We further performed a sensitivity analysis to adjust for several confounders on the effects of dietary treatment on bacterial abundances, including, age, sex, BMI, endoscopy procedure, smoking status, and baseline dietary factors (intakes of energy, fibre, and alcohol). After adjusting for confounders and multiple testing (FDR>0.05), we did not detect any effect of dietary supplements on microbiota abundances. Results were consistent when using the MaAsLin2 R package^(29)^ to conduct mixed effect modelling; no significant associations were identified after adjusting for participant ID as a random effect, and the covariates listed above as fixed effects.

### Inter-individual response to intervention

Although no effects of the intervention on gut microbiome diversity was detected for the study participants as a whole, Figure 2 and Figure 3 show relatively large, inter-individual changes in diversity metrics. To identify participants who displayed consistent gut microbiota responses to the intervention, we performed DMM clustering. Microbiome profiles were clustered into three microbial ‘enterotypes’ (Supplementary Figure 2). Cluster 1 was composed mostly of *Bacteroides, Faecalibacterium*, and unclassified Ruminococcaceae. Cluster 2 was driven by high relative abundances of *Bacteroides, Faecalibacterium*, and unclassified Lachnospiraceae, while cluster 3 was driven by high relative abundance of *Prevotella, Faecalibacterium*, and *Bacteroides* abundance.

**Figure 5:**
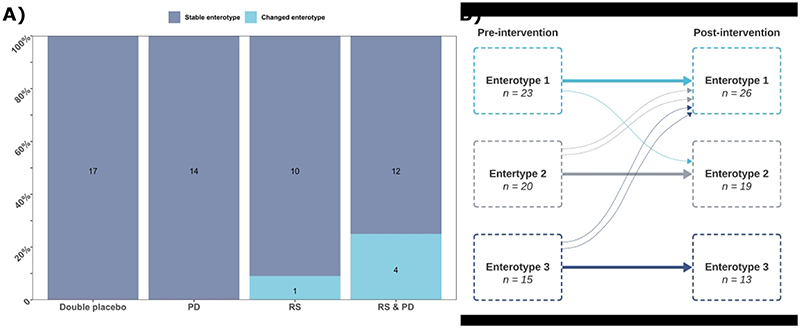
Dirichlet’s multinomial mixture clustering of participants. **A)** Proportional bar plot summarising the number of participants whose enterotype was unchanged and those who changed gut microbial enterotype post-intervention. **B)** Movement of participants between the identified clusters (enterotypes) between pre- and post-intervention. Thin lines represent individual participants. Abbreviations: PD, polydextrose; RS, resistant starch.

In response to the intervention, most participants remained in their baseline enterotype (Figure 5). However, five participants transitioned from one enterotype to another, four from the RS + PD arm and one participant from the RS arm (Figure 5A). The participant from the RS arm that transitioned enterotypes went from enterotype 2 pre-intervention to enterotype 1 post-intervention. Two participants from the RS + PD group transitioned from enterotype 3 pre-intervention to enterotype 1 post-intervention; one participant went from enterotype 1 to enterotype 2, and one participant transitioned from enterotype 2 to enterotype 1 (Figure 5B). The numbers switching enterotypes were not statistically significant between groups.

### Effects of resistant starch and polydextrose on metabolite concentrations in stool, plasma, and urine

The results for the effects of RS and PD on faecal, plasma, and urinary metabolite concentrations, including SCFAs and branched-chain fatty acids (BCFAs), are displayed in Table 2. Participants supplemented with PD had lower post-intervention faecal concentrations of isobutyrate (LSM (SD) 1.28 (0.12) PD vs. 1.67 (0.13) placebo, p=0.042) and of valeric acid (LSM (SD) 1.76 (0.17) PD vs. 2.48 (0.17) placebo, p=0.006). There were no effects of RS or PD, or an interaction between the two NDCs, on the other faecal, plasma, and urinary microbial metabolites measured.

When using delta change in metabolite concentrations between baseline and post-intervention as the outcome, and after adjusting for age, sex, BMI, procedure type, smoking status, baseline dietary factors (energy, fibre, and alcohol) and multiple testing, we noted that PD elevated faecal butyrate concentrations following the intervention (β[95% CI] = 5.15 [0.55: 9.76], *p*_*adj*_=0.03).

We further investigated the correlations between metabolite concentrations derived from stool, plasma, and urine samples pre- and post-intervention. We detected multiple significant intra-sample correlations between microbial metabolites; however, inter-sample correlations were scarcer (Supplementary Figure 3). Of note, there were relatively strong negative correlations between urinary acetate concentrations and faecal concentrations of isovaleric acid and 2-methyl-1-butanol at baseline (pre-intervention).

### Associations between gut microbiota and short-chain fatty acid concentrations

We further explored the relationship between changes in gut microbiota abundance and changes in faecal SCFA concentrations post-intervention. After adjusting for potential confounders, we detected possible associations between changes in gut microbial taxa, including *Hydrogenoanaerobacterium, Roseburia* and *Collinsella*, and changes in acetate (Supplementary Figure 4), propionate (Supplementary Figure 5), and butyrate concentrations (Supplementary Figure 6). However, none of these associations reached statistical significance following correction for multiple testing (FDR <0.05).

## Discussion

To our knowledge, the DISC Study is the first study to investigate the effects of both RS and PD, or their combination, on the diversity and composition of the gut microbiota, as well as on faecal, plasma, and urinary metabolite concentrations, particularly SCFAs, in healthy participants. Further, to our knowledge, this is the largest RCT in which healthy participants have been supplemented with PD, and second largest which has supplemented with RS^(7)^ – the study by Alfa et al. included 84 participants^(30)^. The DISC Study also had the longest duration of supplementation with PD^(7)^, and second longest to supplement with RS (the longest trial duration being 72 days^(30)^).

After adjusting for multiple testing and for potential confounders, we observed no effects of supplementation with RS and/or PD for 50 days on alpha-diversity, beta-diversity, or on the relative abundance of bacteria phyla and genera. However, using clustering-based approaches that grouped individuals into enterotypes, we detected shifts in composition of the gut microbiota for some individuals in response to the RS and RS & PD intervention. This includes one participant supplemented with RS only and four participants supplemented with RS + PD (Figure 5). Importantly, individuals randomised to the double placebo or PD arm did not change enterotype. This suggests that both the type and amount of dietary fibre may influence responses to intervention with larger amounts of dietary fibre (RS + PD) provoking enterotype change in more participants. The findings are in line with our earlier results on markers of colorectal cancer risk in the same cohort, where an effect of RS only on crypt cell proliferation was observed^(14)^.

In addition, the type of RS administered may be important in determining effects on the gut microbiome. Martinez et al. investigated the effects of supplementation with either 33g per day of RS type 2 (a native granular starch consisting of ungelatinized granules) or type 4 (chemically-modified starch) in 10 healthy participants over 3 weeks and noted significant differences in their effects on human faecal microbiota composition^(31)^. The RS type 4 (given as Fibersym^®^) significantly decreased Firmicutes and increased Bacteroidetes and Actinobacteria, whereas RS type 2 (given as Hi-Maize 260) produced no changes at the phylum level^(31)^. In agreement with the findings from the present study, which used RS type 2, they found no effects of either type of RS on alpha-diversity ^(31)^. In contrast, in a 3-months long RCT in which 82 healthy participants were supplemented with a potato-derived RS type 2 or corn starch placebo, there were significant effects on two measures of alpha-diversity (Shannon diversity index and inverse Simpson index) ^(30)^. RS type 2, used in the DISC Study, is present in intact starch granules that can be found in foods such as bananas (4-5g/100g in ripe bananas and ∼18g/100g in green, unripe bananas ^(32)^) ^(33)^.

Only four other RCTs have explored the effects of supplementing healthy adults with PD on the gut microbiota^(34; 35; 36; 37)^. Boler and colleagues examined the effects of supplementation with PD compared with soluble maize fibre or no fibre control on three specific faecal bacterial species and reported significant differences in post-intervention abundance of *Bifidobacterium* spp., *Lactobacillus* spp, and *Escherichia coli* in those given PD^(35)^. In a placebo-controlled, double-blind, crossover study of 31 participants, 8 grams of PD daily for three weeks significantly increased alpha-diversity (Simpson’s index) and the abundance of butyrate-producing *Ruminococcus intestinalis* and bacteria of the *Clostridium* clusters I, II and IV^(34)^. These changes were associated with lower faecal water genotoxicity, suggesting a reduction in risk factors that may be associated with early stages of colorectal carcinogenesis^(34)^. There were no effects of supplementation with PD on faecal SCFA concentrations^(34)^. Beards and colleagues supplemented 40 healthy participants with chocolate products containing different blends of sucrose replacers (RS, PD, or maltitol) for six weeks and found a significant increase in numbers of *lactobacilli, Clostridium histolyticum/perfringens* populations, Bacteroides, *E. rectale, R. flavefaciens*, and total bacteria^(36)^. Over the six-week period, there was also a significant increase in faecal acetate, propionate, and butyrate concentrations^(36)^. In healthy Chinese adults, supplementation with PD for 28 days increased *Lactobacillus* and *Bifidobacterium* species and decreased *Bacteroides* species^(37)^. Higher doses of PD increased faecal concentrations of acetate, butyrate and isobutyrate, and, perhaps surprisingly, resulted in higher colonocyte proliferation^(37)^. In contrast, in 21 healthy adult men with a low habitual dietary fibre intake (∼13-15g/d), supplementation with 21g/d of PD for 21 days resulted in lower faecal acetate, propionate, and butyrate concentrations compared with soluble maize fibre or no fibre control^(35)^. In agreement with the findings of Boler and colleagues^(35)^, in the DISC Study lower faecal concentrations of butyrate and valeric acid SCFAs, and of the BCFA isobutyrate, were observed in participants supplemented with PD compared with placebo. We did not observe significant effects of RS, compared with placebo, on faecal, urinary or plasma SCFA. Although there is evidence that RS is one of the most effective dietary fibres at modulating the gut microbiota to stimulate butyrate production^(38)^, our systematic dose-response studies in the rat model have shown that the extent to which SCFA patterns are driven towards high molar proportion of butyrate depends on RS dose with lower molar proportions of butyrate observed with higher RS doses^(39)^. Importantly, in humans, the effects of RS on large bowel SCFA patterns vary at the individual participant level^(40; 41)^ and some studies have reported no effects on butyrate concentrations^(42)^. In addition, the effects of RS on butyrate, and other SCFA, are influenced by RS type^(43)^. For example, although potato dish supplementation (providing RS type 2 and/or 3) for four weeks increased the abundance of butyrate-producing *Roseburia faecis* in healthy adults, there were no significant effects on faecal SCFA concentrations^(44)^. When healthy young adults were supplemented with different types of RS for 2 weeks, only that from potatoes, but not RS from maize, increased faecal butyrate concentrations^(41)^.

The lack of consistency in observations between the present study and some other studies of RS and PD supplementation in humans reflects the heterogeneity in findings reported in the systematic review and meta-analysis of effects of dietary fibre on the gut microbiome by So and colleagues^(7)^. In addition to heterogeneity in characteristics of the study participants, this lack of consistency in response to supplementation may be explained by several factors related to the protocols for the intervention studies with the most prominent differences being type and dose of dietary fibre, study duration, comparator treatment, and study design (most studies to date have been crossover trials)^(45)^. For example, RS type 4 may lead to larger changes in the composition of the gut microbiota than other RS types^(31)^. Between study differences in findings may also be due to differences in the methods used to analyse the gut microbiota and to quantify concentrations of SCFA and other microbial metabolites.

### Strengths and Limitations

A strength of this study is that it was a RCT that investigated the effects of equal doses of two contrasting types of dietary fibres (RS and PD) given for 50 days, which is one of the largest and longest duration studies of the effects of dietary fibre on the gut microbiome^(7)^. The doses used (12g of PD and 23g RS per day) were designed to provide similar quantities of additional dietary fibre i.e. 11 - 12g per day. As adults (aged 19-64) in the UK are estimated to consume, on average, 19.7g of dietary fibre (AOAC definition) per day^(46)^, the doses given would enhance fibre intake to the recommended level i.e. 30g/d^(47)^. Further, the ingredients were chosen as intervention agents because they can be incorporated into foods conveniently to enhance dietary fibre intake. Although RS and PD did not change the composition of the gut microbiota in this study, dietary fibre has a range of other beneficial effects including improving gut motility and contributing to metabolic health. Gastrointestinal transit time is a major modulator of the gut microbiome and of microbial metabolites both *in vitro*^(48)^ and *in vivo*^(49)^. Whilst some dietary fibres are impact gastrointestinal transit time^(50; 51; 52)^, there is much less evidence for a consistent effect of RS on transit time^(39; 53; 54; 55)^. Since we did not observe significant effects of RS or PD on the measured outcomes, we do not anticipate any effects on transit time.

We also performed sensitivity analyses to adjust for several potential confounders i.e., age, sex, endoscopy procedure at baseline, baseline dietary fibre intake, and BMI. The results from these sensitivity analyses were comparable to those from our initial analyses, confirming our findings.

Another strength is that, in addition to characterising changes in the gut microbiota, we investigated effects on SCFAs and other microbial metabolites in three different sample types i.e., stool, plasma, and urine. However, faecal concentrations of SCFA may not be fully representative of intraluminal SCFAs. For example, autopsy study of sudden death victims showed that concentrations of SCFA fell from the proximal to the distal colon^(56)^. However, concentration of total SCFA in faeces were similar to those in the sigmoid and rectum^(56)^.

A limitation of our study is that it may have been underpowered given that *a priori* sample size calculations were not conducted and that this was a secondary analysis using available data and samples. Although the DISC Study included 75 participants, for the current investigation, paired baseline and follow-up data were available for a subset of 58 participants only which limited our statistical power to detect effects of the intervention. That said, this is one of the largest randomised controlled studies on the effects of RS and PD on gastrointestinal microbiota and their metabolites in humans. Further, given the considerable inter-individual variation observed both at baseline and in response to the dietary fibre supplementation, the study duration and size may not have been sufficient to detect intervention effects at a group level.

Another limitation of this study is that dietary data were self-reported, which can introduce self-report bias^(57)^. The mean habitual dietary fibre intake at baseline in our study was 22.5g per day (mean intake by adults in the UK is approximately 18g/d) which may suggest recruitment of participants with relatively healthier diets, despite the majority of participants having obesity, or an over-estimation of dietary fibre intake. We asked participants to maintain their habitual diet, but changes in other lifestyle factors such as physical activity, with potential to influence the gut microbiota and/or their metabolism, may have occurred during the intervention period. Lastly, all 58 participants in this study identified as White British, restricting the generalisability of our findings to wider, more ethnically diverse populations.

### Conclusions

In the DISC Study, supplementation with RS and/or PD for 50 days did not elicit changes in the gut microbiota of healthy adults. Larger and longer duration studies that supplement with higher doses of dietary fibre are required to further investigate the effects of dietary fibre on the gut microbiome and associated metabolites. Such studies should focus on the effects of different fibre subtypes (e.g., RS type 1 vs. RS type 2), be conducted in participants with different health status (e.g., individuals with bacterial dysbiosis or patients with type 2 diabetes^(58)^ may be more response to dietary intervention), and use alternate study designs, including repeated measures and cross-over studies.

## Supplementary Material

Supplementary Material

## Figures and Tables

**Table 1:** Characteristics of participants in the DISC Study

		Intervention group			
	All	Placebo	PD	RS	RS & PD
***n***	58	17	14	11	16
**RS**		**-**	**-**	**+**	**+**
**PD**		**-**	**+**	**-**	**+**
**Females (n (%))**	32 (55)	7 (41)	10 (71)	9 (82)	6 (28)
**Age (years)**	52.8 (11.9)	50.6 (11.3)	58.8 (15.3)	53.7 (7.0)	51.3 (9.8)
**Ethnicity (n)**					
White British	58 (100)	17 (100)	14 (100)	11 (100)	16 (100)
**Endoscopy procedure (%)**					
Colonoscopy	17 (29)	5 (29)	4 (29)	4 (36)	4 (25%)
Flexible sigmoidoscopy	41 (71)	12 (71)	10 (71)	7 (64)	12 (75%)
**BMI (kg/m^2^)**	30.3 (5.4)	30.6 (4.6)	29.3 (7.1)	30.7 (4.8)	29.9 (5.8)
**Smoking status (n)**					
Never	29 (50)	10 (59)	7 (50)	6 (55)	6 (28)
Former	15 (26)	3 (18)	4 (29)	3 (27)	5 (31)
Current	14 (24)	4 (24)	3 (21)	2 (18)	5 (31)
**Dietary fibre** **intake (g/day)**	22.5 (10.9)	23.0 (12.7)	21.7 (9.7)	21.5 (13.8)	23.9 (13.5)

Data are presented as number and proportion of participants (*n* (%)) with the exception of age, BMI, and dietary fibre intake presented as mean (SD). Dietary fibre estimated using Englyst method.

*Abbreviations: RS, resistant starch; PD, polydextrose; BMI, body mass index*.

**Table 2:** Effects of RS and PD on post-intervention microbial metabolite concentrations in stool, plasma, and urine

Microbial metabolite	Effect of RS			Effect of PD			P interaction
	-	+	P value	-	+	P value	
*Stool*							
**Acetate** **(μMol/mL)**	41.5 (3.6)	50.1 (3.9)	0.112	48.6 (3.8)	43.0 (3.6)	0.300	0.306
**Propionate** **(μMol/mL)**	13.6 (1.4)	14.0 (1.5)	0.868	14.1 (1.5)	13.6 (1.4)	0.805	0.303
**Butyrate** **(μMol/mL)**	12.7 (1.4)	14.9 (1.5)	0.304	15.8 (1.5)	11.8 (1.4)	0.055	0.139
**Isobutyrate** **(μMol/mL)**	1.51 (0.12)	1.43 (0.13)	0.653	1.67 (0.13)	1.28 (0.12)	0.042*	0.264
**2MB** **(μMol/mL)**	0.96 (0.09)	0.84 (0.09)	0.378	1.00 (0.09)	0.80 (0.09)	0.115	0.488
**Valeric acid** **(μMol/mL)**	1.98 (0.17)	2.25 (0.18)	0.268	2.48 (0.18)	1.76 (0.17)	0.006*	0.880
**Isovaleric acid** **(μMol/mL)**	1.18 (0.11)	1.10 (0.11)	0.506	1.28 (0.11)	0.97 (0.11)	0.053	0.232
**Lactic acid** **(μMol/mL)**	1.14 (0.08)	1.09 (0.08)	0.721	1.09 (0.08)	1.14 (0.08)	0.719	0.176
*Plasma*							
**Acetate** **(μMol/L)**	103.1 (6.9)	101.1 (7.6)	0.848	100.9 (7.5)	103.4 (7.5)	0.810	0.895
**Propionate** **(μMol/L)**	7.9 (1.1)	5.8 (1.2)	0.202	6.7 (1.2)	7.0 (1.2)	0.842	0.192
**Butyrate** **(μMol/L)**	0.72 (0.18)	0.98 (0.19)	0.316	0.85 (0.19)	0.85 (0.19)	0.998	0.180
**Isobutyrate** **(μMol/L)**	2.37 (0.41)	2.27 (0.45)	0.877	2.20 (0.44)	2.43 (0.42)	0.710	0.463
**2MB** **(μMol/L)**	3.87 (0.71)	3.98 (0.78)	0.918	3.82 (0.78)	4.03 (0.74)	0.846	0.743
**SCFA** **(μMol/L)**	110.2 (7.5)	109.4 (8.2)	0.943	105.8 (8.1)	113.7 (7.7)	0.487	0.857
**BCFA** **(μMol/L)**	11.1 (2.0)	11.8 (2.2)	0.807	11.2 (2.1)	11.7 (2.0)	0.871	0.838
**VFA** **(μMol/L)**	121.4 (7.8)	121.2 (8.6)	0.988	117.3 (8.5)	125.3 (8.1)	0.508	0.904
*Urine*							
**Acetate** **(μMol/L)**	137.9 (29.7)	95.8 (32.1)	0.344	115.3 (32.1)	118.4 (30.3)	0.946	0.313
**Propionate** **(μMol/L)**	4.14 (0.49)	3.54 (0.52)	0.415	4.40 (0.52)	3.28 (0.49)	0.132	0.199
**Butyrate** **(μMol/L)**	3.03 (0.69)	2.33 (0.75)	0.501	3.48 (0.74)	1.88 (0.70)	0.129	0.185
**Isobutyrate** **(μMol/L)**	2.02 (0.30)	2.05 (0.33)	0.961	2.35 (0.32)	1.72 (0.31)	0.171	0.205
**2MB** **(μMol/L)**	2.69 (0.48)	2.63 (0.52)	0.930	2.71 (0.52)	2.61 (0.49)	0.893	0.345
**SCFA** **(μMol/L)**	144.9 (30.1)	101.8 (32.5)	0.338	123.1 (32.5)	123.7 (30.6)	0.990	0.299
**BCFA** **(μMol/L)**	6.15 (0.91)	5.89 (0.98)	0.844	6.58 (0.98)	5.45 (0.92)	0.410	0.197
**VFA** **(μMol/L)**	151.0 (30.1)	107.8 (32.6)	0.339	129.5 (32.6)	129.2 (30.7)	0.995	0.286

Data are presented as least squares means (LSMs) for post-intervention data adjusted for pre-intervention measurement, age, sex, endoscopy procedure, BMI, and smoking status (ANOVA GLM). Standard error of the mean (SEM) are included in parentheses.

* Significant effect of the intervention (*p*<0.05). P interaction: P value for interaction effect of RS*PD.

*Abbreviations: 2MB, 2-methylbutyrate;* BCFA, branched-chain fatty acids; PD, polydextrose; RS, resistant starch; SCFA, short-chain fatty acids; VFA, volatile fatty acids.

## Data Availability

Metadata are available at: Malcomson, FC (2023), “DISC Study Microbiota and SCFA data”, Mendeley Data, V2, DOI:10.17632/859pyzsxsg

## References

[1] McKeown NM, Fahey GC, Slavin J (2022). Fibre intake for optimal health: how can healthcare professionals support people to reach dietary recommendations?. BMJ.

[2] Reynolds A, Mann J, Cummings J (2019). Carbohydrate quality and human health: a series of systematic reviews and meta-analyses. Lancet.

[3] Cummings JH, Stephen AM (2007). Carbohydrate terminology and classification. Eur J Clin Nutr.

[4] Stowell JD, Rastall DCaRA (2009). Prebiotics and Probiotics Science and Technology.

[5] Raigond P, Dutt S, Singh B, Mérillon J-M, Ramawat KG (2019). Bioactive Molecules in Food.

[6] Swanson KS, Gibson GR, Hutkins R (2020). The International Scientific Association for Probiotics and Prebiotics (ISAPP) consensus statement on the definition and scope of synbiotics. Nat Rev Gastroenterol Hepatol.

[7] So D, Whelan K, Rossi M (2018). Dietary fiber intervention on gut microbiota composition in healthy adults: a systematic review and meta-analysis. Am J Clin Nutr.

[8] Canani RB, Costanzo MD, Leone L (2011). Potential beneficial effects of butyrate in intestinal and extraintestinal diseases. World J Gastroenterol.

[9] Chambers ES, Byrne CS, Morrison DJ (2019). Dietary supplementation with inulin-propionate ester or inulin improves insulin sensitivity in adults with overweight and obesity with distinct effects on the gut microbiota, plasma metabolome and systemic inflammatory responses: a randomised cross-over trial. Gut.

[10] Chambers ES, Viardot A, Psichas A (2015). Effects of targeted delivery of propionate to the human colon on appetite regulation, body weight maintenance and adiposity in overweight adults. Gut.

[11] Rios-Covian D, Gonzalez S, Nogacka AM (2020). An Overview on Fecal Branched Short-Chain Fatty Acids Along Human Life and as Related With Body Mass Index: Associated Dietary and Anthropometric Factors. Front Microbiol.

[12] Boler BM, Serao MC, Bauer LL (2011). Digestive physiological outcomes related to polydextrose and soluble maize fibre consumption by healthy adult men. Br J Nutr.

[13] Malcomson FC, Willis ND, McCallum I (2017). Effects of supplementation with nondigestible carbohydrates on fecal calprotectin and on epigenetic regulation of SFRP1 expression in the large-bowel mucosa of healthy individuals1,2. The American Journal of Clinical Nutrition.

[14] Malcomson FC, Willis ND, McCallum I (2020). Resistant starch supplementation increases crypt cell proliferative state in the rectal mucosa of older healthy participants. Br J Nutr.

[15] Malcomson FC, Willis ND, McCallum I (2017). Non-digestible carbohydrates supplementation increases miR-32 expression in the healthy human colorectal epithelium: A randomized controlled trial. Mol Carcinog.

[16] Kroke A, Klipstein-Grobusch K, Voss S (1999). Validation of a self-administered food-frequency questionnaire administered in the European Prospective Investigation into Cancer and Nutrition (EPIC) Study: comparison of energy, protein, and macronutrient intakes estimated with the doubly labeled water, urinary nitrogen, and repeated 24-h dietary recall methods. Am J Clin Nutr.

[17] Kozich JJ, Westcott SL, Baxter NT (2013). Development of a dual-index sequencing strategy and curation pipeline for analyzing amplicon sequence data on the MiSeq Illumina sequencing platform. Appl Environ Microbiol.

[18] Rognes T, Flouri T, Nichols B (2016). VSEARCH: a versatile open source tool for metagenomics. PeerJ.

[19] Peuranen S, Tiihonen K, Apajalahti J (2004). Combination of polydextrose and lactitol affects microbial ecosystem and immune responses in rat gastrointestinal tract. Br J Nutr.

[20] Morrison DJ, Cooper K, Waldron S (2004). A streamlined approach to the analysis of volatile fatty acids and its application to the measurement of whole-body flux. Rapid Commun Mass Spectrom.

[21] Dronamraju SS, Coxhead JM, Kelly SB (2009). Cell kinetics and gene expression changes in colorectal cancer patients given resistant starch: a randomised controlled trial. Gut.

[22] R Core Team (2020). R: A language and environment for statistical computing.

[23] Alkek Center for Metagenomics and Microbiome Research (2019). ATIMA (Agile toolKit for Inclusive Microbial Analyses).

[24] Benjamini Y, Hochberg Y (1995). Controlling the False Discovery Rate: A Practical and Powerful Approach to Multiple Testing. Journal of the Royal Statistical Society Series B (Methodological).

[25] Holmes I, Harris K, Quince C (2012). Dirichlet Multinomial Mixtures: Generative Models for Microbial Metagenomics. Plos One.

[26] Bliss CI, Greenwood ML, White ES (1956). A Rankit Analysis of Paired Comparisons for Measuring the Effect of Sprays on Flavor. Biometrics.

[27] Englyst HN, Quigley ME, Hudson GJ (1994). Determination of dietary fibre as non-starch polysaccharides with gas-liquid chromatographic, high-performance liquid chromatographic or spectrophotometric measurement of constituent sugars. Analyst.

[28] King CH, Desai H, Sylvetsky AC (2019). Baseline human gut microbiota profile in healthy people and standard reporting template. PLOS ONE.

[29] Mallick H, Rahnavard A, McIver LJ (2021). Multivariable association discovery in population-scale meta-omics studies. PLOS Computational Biology.

[30] Alfa MJ, Strang D, Tappia PS (2018). A randomized trial to determine the impact of a digestion resistant starch composition on the gut microbiome in older and mid-age adults. Clin Nutr.

[31] Martínez I, Kim J, Duffy PR (2010). Resistant Starches Types 2 and 4 Have Differential Effects on the Composition of the Fecal Microbiota in Human Subjects. Plos One.

[32] Phillips KM, McGinty RC, Couture G (2021). Dietary fiber, starch, and sugars in bananas at different stages of ripeness in the retail market. Plos One.

[33] Mathers JC (2023). Dietary fibre and health: the story so far. Proc Nutr Soc.

[34] Costabile A, Fava F, Röytiö H (2012). Impact of polydextrose on the faecal microbiota: a double-blind, crossover, placebo-controlled feeding study in healthy human subjects. Brit J Nutr.

[35] Boler BMV, Serao MCR, Bauer LL (2011). Digestive physiological outcomes related to polydextrose and soluble maize fibre consumption by healthy adult men. Brit J Nutr.

[36] Beards E, Tuohy K, Gibson G (2010). A human volunteer study to assess the impact of confectionery sweeteners on the gut microbiota composition. Brit J Nutr.

[37] Jie Z, Bang-Yao L, Ming-Jie X (2000). Studies on the effects of polydextrose intake on physiologic functions in Chinese people. Am J Clin Nutr.

[38] Cummings JH, Macfarlane GT, Englyst HN (2001). Prebiotic digestion and fermentation. Am J Clin Nutr.

[39] Mathers JC, Smith H, Carter S (1997). Dose-response effects of raw potato starch on small-intestinal escape, large-bowel fermentation and gut transit time in the rat. Br J Nutr.

[40] Venkataraman A, Sieber JR, Schmidt AW (2016). Variable responses of human microbiomes to dietary supplementation with resistant starch. Microbiome.

[41] Baxter NT, Schmidt AW, Venkataraman A (2019). Dynamics of Human Gut Microbiota and Short-Chain Fatty Acids in Response to Dietary Interventions with Three Fermentable Fibers. mBio.

[42] DeMartino P, Cockburn DW (2020). Resistant starch: impact on the gut microbiome and health. Current Opinion in Biotechnology.

[43] Teichmann J, Cockburn DW (2021). In vitro Fermentation Reveals Changes in Butyrate Production Dependent on Resistant Starch Source and Microbiome Composition. Front Microbiol.

[44] DeMartino P, Johnston EA, Petersen KS (2022). Additional Resistant Starch from One Potato Side Dish per Day Alters the Gut Microbiota but Not Fecal Short-Chain Fatty Acid Concentrations. Nutrients.

[45] Dobranowski PA, Stintzi A (2021). Resistant starch, microbiome, and precision modulation. Gut Microbes.

[46] PHE/FSA (2020). National Diet and Nutrition Survey Rolling programme Years 9 to 11 (2016/2017 to 2018/2019) A survey carried out on behalf of Public Health England and the Food Standards Agency.

[47] SACN (2015). SACN Carbohydrates and Health Report.

[48] Minnebo Y, Delbaere K, Goethals V (2023). Gut microbiota response to in vitro transit time variation is mediated by microbial growth rates, nutrient use efficiency and adaptation to in vivo transit time. Microbiome.

[49] Asnicar F, Leeming ER, Dimidi E (2021). Blue poo: impact of gut transit time on the gut microbiome using a novel marker. Gut.

[50] Muller-Lissner SA (1988). Effect of wheat bran on weight of stool and gastrointestinal transit time: a meta analysis. Br Med J (Clin Res Ed).

[51] Payler DK, Pomare EW, Heaton KW (1975). The effect of wheat bran on intestinal transit. Gut.

[52] Muir JG, Yeow EG, Keogh J (2004). Combining wheat bran with resistant starch has more beneficial effects on fecal indexes than does wheat bran alone. Am J Clin Nutr.

[53] Cummings JH, Beatty ER, Kingman SM (1996). Digestion and physiological properties of resistant starch in the human large bowel. Br J Nutr.

[54] Tomlin J, Read NW (1990). The effect of resistant starch on colon function in humans. Br J Nutr.

[55] Sharma A, Yadav BS, Ritika (2008). Resistant Starch: Physiological Roles and Food Applications. Food Reviews International.

[56] Cummings JH, Pomare EW, Branch WJ (1987). Short chain fatty acids in human large intestine, portal, hepatic and venous blood. Gut.

[57] Penn L, Boeing H, Boushey CJ (2010). Assessment of dietary intake: NuGO symposium report. Genes Nutr.

[58] Ojo O, Ojo OO, Zand N (2021). The Effect of Dietary Fibre on Gut Microbiota, Lipid Profile, and Inflammatory Markers in Patients with Type 2 Diabetes: A Systematic Review and Meta-Analysis of Randomised Controlled Trials. Nutrients.

