# Effects of non-digestible carbohydrates on gut microbiota and microbial metabolites: a randomised, controlled dietary intervention in healthy individuals

**DOI:** 10.1017/S000711452400271X

**Published:** 2024-12-14

**Authors:** Fiona C. Malcomson, Panayiotis Louca, Andrew Nelson, Naomi D. Willis, Iain McCallum, Long Xie, Arthur C. Ouwehand, Julian D. Stowell, Tom Preston, Douglas J. Morrison, Seamus B. Kelly, D. Michael Bradburn, Nigel J. Belshaw, Ian T. Johnson, Bernard M. Corfe, Christopher J. Stewart, John C. Mathers

**Affiliations:** 1 Human Nutrition and Exercise Research Centre, Centre for Healthier Lives, Population Health Sciences Institute, Newcastle University, Newcastle upon Tyne NE2 4HH, UK; 2 Centre for Cancer, Population Health Sciences Institute, Newcastle University, Newcastle upon Tyne, UK; 3 Department of Applied Science, Northumbria University, Newcastle upon Tyne NE1 8ST, UK; 4 Northumbria Healthcare NHS Foundation Trust, North Tyneside General Hospital, Rake Lane, North Shields NE29 8NH, UK; 5 International Flavors & Fragrances, Kantvik 02460, Finland; 6 Sabri Ülker Foundation, Istanbul, Turkey; 7 Scottish Universities Environmental Research Centre, College of Science and Engineering, University of Glasgow, Glasgow, UK; 8 Northumbria Healthcare National Health Service Foundation Trust, Ashington, UK; 9 University of East Anglia, Norwich Research Park, Norwich NR4 7TJ, UK; 10 Quadram Institute, Norwich Research Park, Norwich, Norfolk NR4 7UQ, UK; 11 Translational and Clinical Research Institute, Newcastle University, Newcastle upon Tyne NE2 4HH, UK

**Keywords:** Non-digestible carbohydrates, Resistant starch, Polydextrose, Gut microbiota, SCFA, Randomised controlled trial, Dietary intervention, Humans

## Abstract

The gut microbiome is impacted by certain types of dietary fibre. However, the type, duration and dose needed to elicit gut microbial changes and whether these changes also influence microbial metabolites remain unclear. This study investigated the effects of supplementing healthy participants with two types of non-digestible carbohydrates (resistant starch (RS) and polydextrose (PD)) on the stool microbiota and microbial metabolite concentrations in plasma, stool and urine, as secondary outcomes in the Dietary Intervention Stem Cells and Colorectal Cancer (DISC) Study. The DISC study was a double-blind, randomised controlled trial that supplemented healthy participants with RS and/or PD or placebo for 50 d in a 2 × 2 factorial design. DNA was extracted from stool samples collected pre- and post-intervention, and V4 16S rRNA gene sequencing was used to profile the gut microbiota. Metabolite concentrations were measured in stool, plasma and urine by high-performance liquid chromatography. A total of fifty-eight participants with paired samples available were included. After 50 d, no effects of RS or PD were detected on composition of the gut microbiota diversity (alpha- and beta-diversity), on genus relative abundance or on metabolite concentrations. However, Drichlet’s multinomial mixture clustering-based approach suggests that some participants changed microbial enterotype post-intervention. The gut microbiota and fecal, plasma and urinary microbial metabolites were stable in response to a 50-d fibre intervention in middle-aged adults. Larger and longer studies, including those which explore the effects of specific fibre sub-types, may be required to determine the relationships between fibre intake, the gut microbiome and host health.

Dietary fibre has been defined as carbohydrate polymers with three or more monomeric units that are not hydrolysed by endogenous enzymes in the small intestine^(1)^. It reaches the large intestine and is fermented by resident gut bacteria to produce microbial metabolites such as short-chain fatty acids (SCFA) including acetate, propionate and butyrate. Observational data suggest that higher intakes of dietary fibre, when compared with the lowest consumers, are associated with reduced risk of non-communicable diseases, including colorectal cancer, diabetes and CVD^(2)^. The proposed mechanisms through which dietary fibres exert their beneficial health effects include modulation of the gut microbiota and synthesis of microbial metabolites, primarily SCFA.

Dietary fibres include a wide range of non-digestible carbohydrates that differ in terms of physiochemical properties such as solubility and physiological effects such as fermentability^(1)^. These non-digestible carbohydrates include resistant starch (RS; the sum of starch and products of starch digestion that are not absorbed in the small bowel^(3)^) and polydextrose (PD; a synthetic, soluble fibre and glucose polymer with sorbitol end groups developed as a low-energy sweetener with bulking properties^(4)^). Further, five subtypes of RS exist according to the factors affecting their resistance to digestion in the colon: type 1 (physically inaccessible e.g. wholegrains), type 2 (ungelatinised resistant granules with type B crystallinity e.g. high-amylose maize starch, green bananas), type 3 (retrograded starch e.g. cooked then cooled potatoes), type 4 (chemically modified starches e.g. cross-linked starch in thickeners) and type 5 (amylose–lipid complexes, e.g. palmitic acid amylose complex, foods with high amylose content)^(5)^. Some highly fermentable fibres, including fructooligosaccharides and inulin, exert prebiotic effects, i.e. selectively stimulate the growth or activity of one or a limited number of gut bacteria, and thus confer beneficial effects on host health^(6)^. A recent systematic review and meta-analysis, spanning sixty-four studies and over 2000 participants, investigated the effects of dietary fibre interventions on gut microbiota composition in healthy adults and included eight randomised controlled studies which supplemented with RS and/or PD^(7)^. In the meta-analysis, six studies reported Shannon diversity index and three reported total number of observed operational taxonomic units and showed no overall effect of dietary fibre supplementation on *α*-diversity compared with placebo or low-fibre comparators^(7)^. Subgroup analysis of the effects of fibres classified as candidate prebiotics (which include RS and PD) on bacterial abundance demonstrated increased *Bifidobacterium* spp., but no effects on *Lactobacillus* spp. (the two most commonly reported taxa), compared with placebo or low-fibre controls^(7)^. It is not only the type of dietary fibre that is important, but fibre subtypes (e.g. RS type 1 *v*. type 2) may have differential effects on the gut microbiota and on host health. The authors also explored effects of dietary fibre interventions on SCFA concentrations (but did not perform fibre type subgroup analyses) and reported an increase in fecal butyrate concentrations after dietary fibre interventions compared with placebo or low-fibre comparators (standard mean difference (95 % CI): 0·24 (0·00, 0·47), *P* = 0·05)^(7)^. However, no effects on total fecal SCFA concentrations were observed^(7)^.

The SCFA butyrate exerts chemoprotective and other health-promoting properties that may be beneficial in the prevention or management of diseases such as colorectal cancer, insulin resistance and hypercholesterolaemia^(8)^. Chambers et al. have also shown that targeted delivery of propionate has positive effects on appetite regulation, body weight maintenance and adiposity, and modulated the gut microbiota and plasma metabolome^(9,10)^. In contrast, higher intakes of dietary fibre, particularly insoluble fibre, have been associated with lower concentrations of fecal branched-chain fatty acids (BCFA)^(11)^, end-products of protein decomposition, which may provide an additional chemoprotective effect as certain metabolites produced during this process are carcinogenic. For example, in healthy male volunteers, supplementation with PD or soluble maize fibre for 21 d reduced the production of putrefactive compounds, including fecal BCFA and ammonia^(12)^.

The aim of this study was to investigate the effects of supplementing healthy participants for 50 d with two types of non-digestible carbohydrates which fall within the definition of dietary fibre (RS: Hi-maize® 260 (Ingredion™, type 2 RS) and/or PD: Litesse® *Ultra*™ (International Flavors & Fragances™ Danisco®)) on the gut microbiota and on microbial metabolite concentrations, including SCFA and BCFA, in stool, plasma and urine in the Dietary Intervention Stem Cells and Colorectal Cancer (DISC) Study. This is a secondary analysis of samples and data from the DISC study. We have reported previously the effects of RS and PD on a range of outcomes including inflammatory markers, WNT pathway-related markers, colorectal crypt cell proliferative state and microRNA expression^(13–15)^.

## Experimental methods

**Figure 1.**
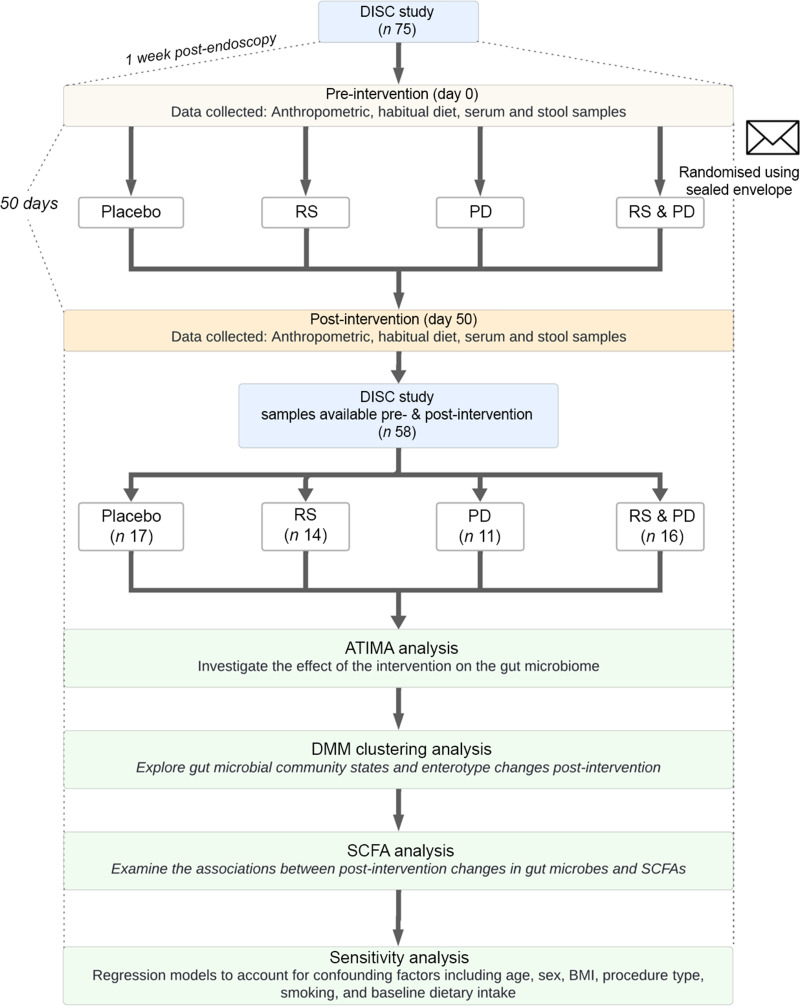
Flow chart of DISC study design and analytical pipeline. ATIMA, Agile Toolkit for Incisive Microbial Analysis; DISC, Dietary Intervention Stem Cells and Colorectal Cancer; DMM, Dirichlet’s multinomial mixture; PD, polydextrose; RS, resistant starch; SCFA, short-chain fatty acids.

A flowchart of the study design and analytical pipeline is presented in Fig. 1.

### The DISC study

The DISC study was a double-blind, randomised, placebo controlled dietary intervention that supplemented seventy-five healthy participants with two types of non-digestible carbohydrates (RS and/or PD) or respective placebos in a 2 × 2 factorial design for 50 d^(13)^. Participants were recruited by the research team from gastroenterology out-patients departments at North Tyneside General Hospital and Wansbeck General Hospital in the North East of England between May 2010 and July 2011 using endoscopy patient lists. Potential participants were sent a study invitation letter with detailed information about the study at least 5 days prior to their hospital appointment. At endoscopy, potential study participants were screened for exclusion criteria, which included: aged < 16 or > 85 years, pregnant or planning to become pregnant, diabetes mellitus, familial adenomatous polyposis syndrome, Lynch syndrome, known colorectal tumour or prior colorectal cancer, prior colorectal resection, active colonic inflammation at endoscopy, iatrogenic perforation at endoscopy, incomplete left-sided examination, colorectal carcinoma discovered at endoscopy or histology, chemotherapy in the last 6 months, administering non-steroidal anti-inflammatories, anti-coagulants or immunosuppressive medication. Ethical approval for the DISC study was granted by the Newcastle and North Tyneside Research Ethics Committee on 10th December 2009 (REC No. 09/H0907/77) and Caldicott approval for the storage of data was provided by the Northumbria NHS Foundation Trust (C1792). All participants provided informed written consent. The clinical trial is registered with ClinicalTrials.gov (Identifier NCT01214681).

Habitual diet was assessed at baseline using a food frequency questionnaire adapted from that used in the EPIC- Norfolk study (version 6, CAMB/PQ/6/1205)^(16)^. Participants were asked to consume their normal diet throughout the study. At least 1 week after their baseline endoscopy procedure, participants were randomised to one of four groups by selecting a sealed, opaque envelope:

- Double placebo (12 g Maltodextrin (RS placebo) + 23 g Amioca starch (PD placebo))
- PD (12 g Litesse® *Ultra*™ (International Flavors & Fragances™ Danisco®)
- RS (23 g Hi-maize® 260, Ingredion™, Food Innovation)
- RS + PD (23 g Hi-maize® 260, Ingredion™, Food Innovation + 12 g Litesse® *Ultra*™, International Flavors & Fragances™ Danisco®)

The allocation codes were locked and participants and the research team were blinded.

The RS utilised in this study was Hi-maize® 260 (Ingredion™), a type 2 RS that is isolated from high-amylose corn hybrids and occurs in the natural granular form and is approximately 53 % RS, with the remaining 40 % comprising digestible starch. PD was given in the form of Litesse® *Ultra*™, a sugar-free soluble fibre developed as a sweetener. It is a glucose polymer produced from sorbitol, dextrose and citric acid, which are derived naturally from corn. The intervention agents were supplied in a white powdered form in foil sachets packed into boxes each containing a week’s worth of sachets. Participants consumed 35 g of intervention supplement per day divided into four sachets (two sachets of each intervention agent). Participants were asked to consume the powdered supplements by adding to cold food or liquids such as yoghurt, orange juice or water. Participants were asked to retain all their sachets, including those that were not consumed. To measure compliance, the number of consumed and not consumed sachets were counted for each participant.

### Sample collection

Participants provided rectal mucosal biopsies, blood, spot urine, stool and buccal cell samples at baseline (day 0) and post-intervention (day 50). Participants who underwent colonoscopy (pre-intervention only) were fasted at the point of blood sample collection. Blood samples were collected in seven 4 ml BD Vacutainer® K3EDTA tubes (Becton Dickinson, UK) and one 5 ml BD Vacutainer® SST™ II Advance tube with gold hemogard closure (Becton Dickinson, UK). EDTA tubes were centrifuged for 5 min at 3100 g and 4°C and plasma extracted and stored at −80°C until analysis.

At the initial endoscopy appointment, participants were provided with equipment for the collection of urine and stool at home. Sample collection was performed prior to the first home visit, seven days post-endoscopy appointment, to minimise any effects of bowel preparation associated with the endoscopy procedures. Post-intervention samples were collected just prior to the participant’s repeat sigmoidoscopy. For each collection, participants were provided with a large sealable bucket pot, a disposable bedpan, two ice packs and a cool bag. Urine and stool samples were kept in cool bags containing ice packs until collected by a member of the research team (pre-intervention samples) or brought to the repeat study visit (post-intervention samples). Stool was processed within 3–18 h of defecation. Samples were divided into 5–6 aliquots and stored immediately at –80°C until analysis.

### Gut microbiota sequencing

Gut microbiota analysis was performed ∼10 years after data/sample collection. DNA was extracted from 250 mg stool samples using the DNeasy® PowerLyzer® PowerSoil® kit (Qiagen, UK) and following the manufacturer’s instructions. Bacterial profiling of the variable region 4 (V4) of the 16S rRNA gene was carried out by NU-OMICS (Northumbria University) based on the Schloss wet-lab MiSeq SOP^(17)^. Briefly, PCR was carried out using 1x Accuprime Pfx Supermix, 0·5 µM each primer and 1 µl of template DNA under the following conditions: 95°C 2 min, 30 cycles 95°C 20 s, 55°C 15 s, 72°C 5 min with a final extension 72°C 10 min. One positive (Zymobiomics Microbial Mock community DNA standard) and one negative control sample were included in each 96-well plate and carried through to sequencing. PCR products were quantified using Quant-iT™ PicoGreen™ dsDNA Assay (Invitrogen), and each sample was normalised to 10 nM and then each 96-well plate was pooled. Each pool was quantified using fragment size determined by BioAnalyzer (Agilent Technologies) and concentration by Qubit (Invitrogen). Pools were combined in equimolar amounts to create a single library then denatured using 0·2N NaOH for 5 min and diluted to a final concentration of 5 pM, supplemented with 25 % PhiX and loaded onto a MiSeq V2 500 cycle cartridge. Fastq files were processed using Mothur (v1·48). Paired reads were merged and then filtered to remove contigs > 275 bp, homopolymer > 8 and any ambiguous base. The high- quality reads were dereplicated and aligned to the Silva reference alignment (v132). Chimeric sequences were removed using VSearch^(18)^ and taxonomy was assigned using the RDP database (v18), and non Bacterial sequences were removed.

### Quantification of microbial metabolite concentrations in stool, urine and plasma

Microbial metabolite analyses were performed within 12–24 months after data/sample collection. The concentrations of microbial metabolites (SCFA, BCFA, volatile fatty acids and lactic acid) in stool were quantified by gas chromatography by International Flavors & Fragances^™^ Danisco^®^, Finland as described previously^(19)^. Briefly, 1 ml of 20 mm pivalic acid and 5 ml of water were added to 1 g of fecal sample, mixed thoroughly and centrifuged at 5000 *g* for 5 min. 250 µl of saturated oxalic acid solution was added to 500 µl of the supernatant, and the mixture was incubated at 48°C for 60 min, before centrifugation at 16 000 *g* for 5 min. The supernatant fraction was used for analysis, with pivalic acid as the internal standard.

Plasma and urinary SCFA and BCFA concentrations were measured by Scottish Universities Environmental Research Centre, East Kilbride, UK, as described by Morrison et al.^(20)^. SCFA and BCFA were extracted and derivatised as tBDMS (tert-butyl dimethyl silyl) esters prior to deuterium dilution analysis by GCMS. An alkaline internal standard mix containing three deuterated SCFA, three deuterated BCFA and 3-methyl valerate was added to each sample. Plasma samples (0·3 ml) were mixed and deproteinised using an ultracentrifuge device (Amicon Ultra 0·5 ml 30 kDa, Merck). Urine samples (1 ml) were spiked with an alkaline internal standard mix, but not ultrafiltered. Both sample types were dried by vacuum centrifuge. Blank tubes, deuterium enriched SCFA standards and unenriched SCFA standards were also dried. Dry samples and standards were acidified with dilute HCl. A volume of methyl tert-butyl ether was added and mixed to extract the SCFA. A sub-sample of the upper methyl tert-butyl ether phase was pipetted into a GC vial, and a composite derivatisation reagent was added. The freshly mixed derivatisation reagent contained tBDMSIM (Merck Sigma, UK) in acetonitrile with a hexanoic acid spike to facilitate removal of an acetate reagent blank. The vials were capped. They were derivatised at 70°C for 60 min and cooled. Samples and standards were analysed by GCMS (Agilent MSD, UK) in selected monitoring mode. SCFA and BCFA concentrations were calculated by deuterium dilution analysis.

### Statistical analyses

The DISC study was not subject to a formal power calculation, and a target of seventy-five participants – allowing for a 10 % dropout rate, was set based on our previous study^(21)^.

Statistical analyses were performed using R version 4.3.1^(22)^, and the Agile Toolkit for Incisive Microbial Analysis (https://atima.research.bcm.edu/) ^(23)^ developed by the Centre for Metagenomics and Microbiome Research at the Baylor College of Medicine. The R-based software, Agile Toolkit for Incisive Microbial Analysis, is a stand-alone tool for analysing and visualising trends in alpha and beta diversity and taxa abundance. The rarefaction depth was set to 13 428, at which all negative controls and sequencing negatives were removed from the dataset. At this depth, 31 % of reads were used (1 879 920/6 097 817 reads retained). Microbial abundances with < 10 % prevalence were removed. Using Agile Toolkit for Incisive Microbial Analysis, Mann–Whitney test was used to compare pre- *v*. post-intervention data according to intervention agent (RS, PD or respective placebos). Differences in beta-diversity (weighted Bray–Curtis distance) were assessed using PERMANOVA, including study ID as a nesting factor. We controlled for false discovery rate (FDR) using the Bejamini-Hochberg procedure^(24)^ and an FDR < 0·05 was considered significant.

To cluster gut microbial community states and distinguish inter-individual variations in gut microbiome response to the intervention we conducted Dirichlet’s multinomial mixture modelling^(25)^. The Laplace approximation criterion was used to determine the optimal number of clusters.

The ANOVA general linear model was used to test for effects of RS and PD and for interactions between the two, on post-intervention SCFA concentrations, adjusting for pre-intervention measurement, age, sex, BMI, endoscopy procedure and smoking status as covariates. Additionally, we calculated and investigated (using the same approaches as above) changes in microbial abundance and SCFA concentrations. Changes in microbial abundance and SCFA concentrations were calculated as delta = post-intervention value – corresponding pre-intervention value. Delta abundances were quantile normalised prior to modelling^(26)^. Confounding variables included age, sex, BMI, procedure type (flexible sigmoidoscopy or colonoscopy), smoking status and baseline dietary factors (intakes of energy, fibre and alcohol). Spearman’s correlations were used to explore metabolite correlations, both pre- and post-intervention, across tissue types (stool, urine and plasma). We also utilised the MaAsLin2 R package to conduct mixed effect modelling and explore the links between gut microbiome composition and intervention response. Within mixed effect modelling, participant ID was modelled as a random effect, intervention and additional covariates were modelled as fixed effects.

## Results

### Participant characteristics

Fifty-eight DISC study participants, for whom paired stool samples (pre- and post-intervention) were available for analyses, were included in this study (Fig. 1), and their characteristics are summarised in Table 1. All participants identified as being White British. The mean age of participants across all groups was 53 (sd 12) years. Just over half of the included participants were female (55 %). Most participants (83 %) were classed as having overweight or obesity based on their BMI. Over two-thirds of participants underwent endoscopic examination by flexible sigmoidoscopy during the baseline (pre-intervention) study visit. Half of the participants were never smokers, 26 % were former smokers and 24 % were current smokers. The mean dietary fibre (assessed using the Englyst method^(27)^) intake at baseline was 22·5 g/d (sd 10·9). At baseline, there were no significant differences between intervention groups in habitual intake of energy (0·987), dietary fibre (*P* = 0·942) or protein (*P* = 0·532).

**Table 1.** Characteristics of participants in the DISC study (Numbers and percentages; mean values and standard deviations)

			Intervention group							
	All		Placebo		PD		RS		RS & PD	
	*n*	%	*n*	%	*n*	%	*n*	%	*n*	%
*n*	58		17		14		11		16	
RS			**–**		**–**		**+**		**+**	
PD			**–**		**+**		**–**		**+**	
Females (*n* (%))	32	55	7	41	10	71	9	82	6	28
Age (years)										
Mean	52·8		50·6		58·8		53·7		51·3	
sd	11·9		11·3		15·3		7·0		9·8	
Ethnicity (*n*)										
White British	58	100	17	100	14	100	11	100	16	100
Endoscopy procedure (%)										
Colonoscopy	17	29	5	29	4	29	4	36	4	25 %
Flexible sigmoidoscopy	41	71	12	71	10	71	7	64	12	75 %
BMI (kg/m^2^)										
Mean	30·3		30·6		29·3		30·7		29·9	
sd	5·4		4·6		7·1		4·8		5·8	
Smoking status (*n*)										
Never	29	50	10	59	7	50	6	55	6	28
Former	15	26	3	18	4	29	3	27	5	31
Current	14	24	4	24	3	21	2	18	5	31
Dietary fibre intake (g/d)										
Mean	22·5		23·0		21·7		21·5		23·9	
sd	10·9		12·7		9·7		13·8		13·5	

RS, resistant starch; PD, polydextrose.

Data are presented as number and proportion of participants (*n* (%)) with the exception of age, BMI and dietary fibre intake presented as mean (sd). Dietary fibre estimated using Englyst method.

### Bacterial profiles in stool at baseline from DISC study participants

Pre-intervention, the bacterial profiles in stool from the DISC study participants reflected those of healthy human adults, as reported by King and colleagues^(28)^; Firmicutes and Bacteroidetes were the most dominant phyla, followed by Proteobacteria, Actinobacteria and Verrucomicrobia (data not shown). At the genus level, the most abundant genera were *Bacteroides, Faecalibacterium, Prevotella, Alistipes* and *Roseburia.* There were no differences in the relative abundance of these bacteria between the four intervention groups at baseline. However, at the genus level, the relative abundance of *Streptococcus* was lower in participants randomised to the double placebo arm (FDR < 0·05).

### Effects of resistant starch and polydextrose on gut microbiota diversity

**Figure 2.**
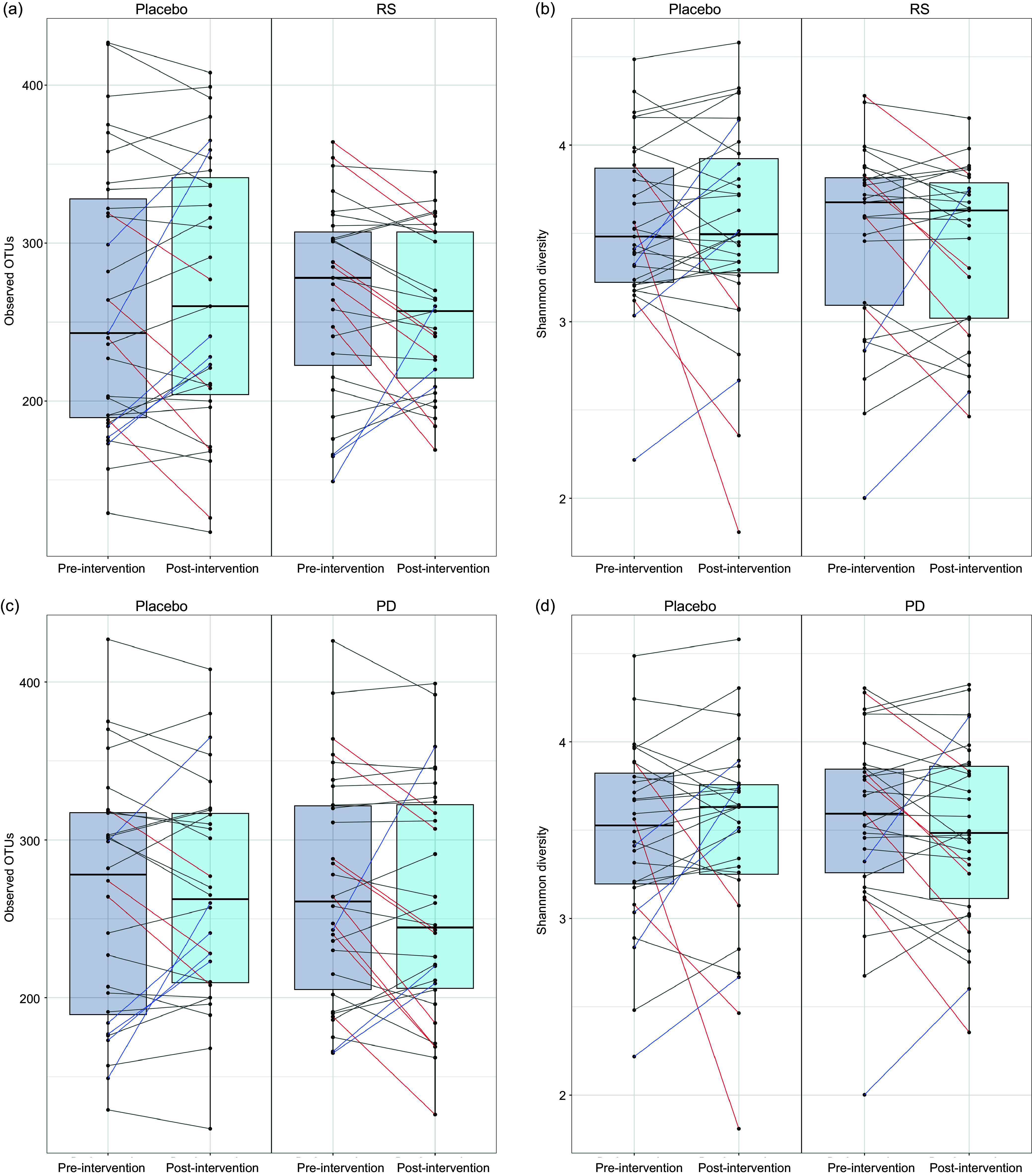
Effects of supplementation with RS and PD on two metrics of alpha-diversity. Alpha-diversity is represented by both observed operational taxonomic units (OTU) and Shannon Diversity. Panels A and B show the impact of RS supplementation on *α* diversity measured by observed OTU and Shannon diversity, respectively. Panels C and D illustrate the effects of PD supplementation on the same *α* diversity metrics. Each point represents individual participants pre- and post-intervention. Lines are coloured if change was > 1 sd, red lines between paired points represent a decrease and blue lines an increase, black lines represent changes of < 1 sd. PD, polydextrose; RS, resistant starch.

When investigating effects of the intervention on alpha-diversity metrics (observed operational taxonomic units (richness) and Shannon index), no effects were detected post-intervention on alpha-diversity for either RS (observed operational taxonomic units, *P* = 0·833; Shannon index, *P* = 0·867) or PD (observed operational taxonomic units, *P* = 0·77; Shannon index, *P* = 0·89) (Fig. 2).

**Figure 3.**
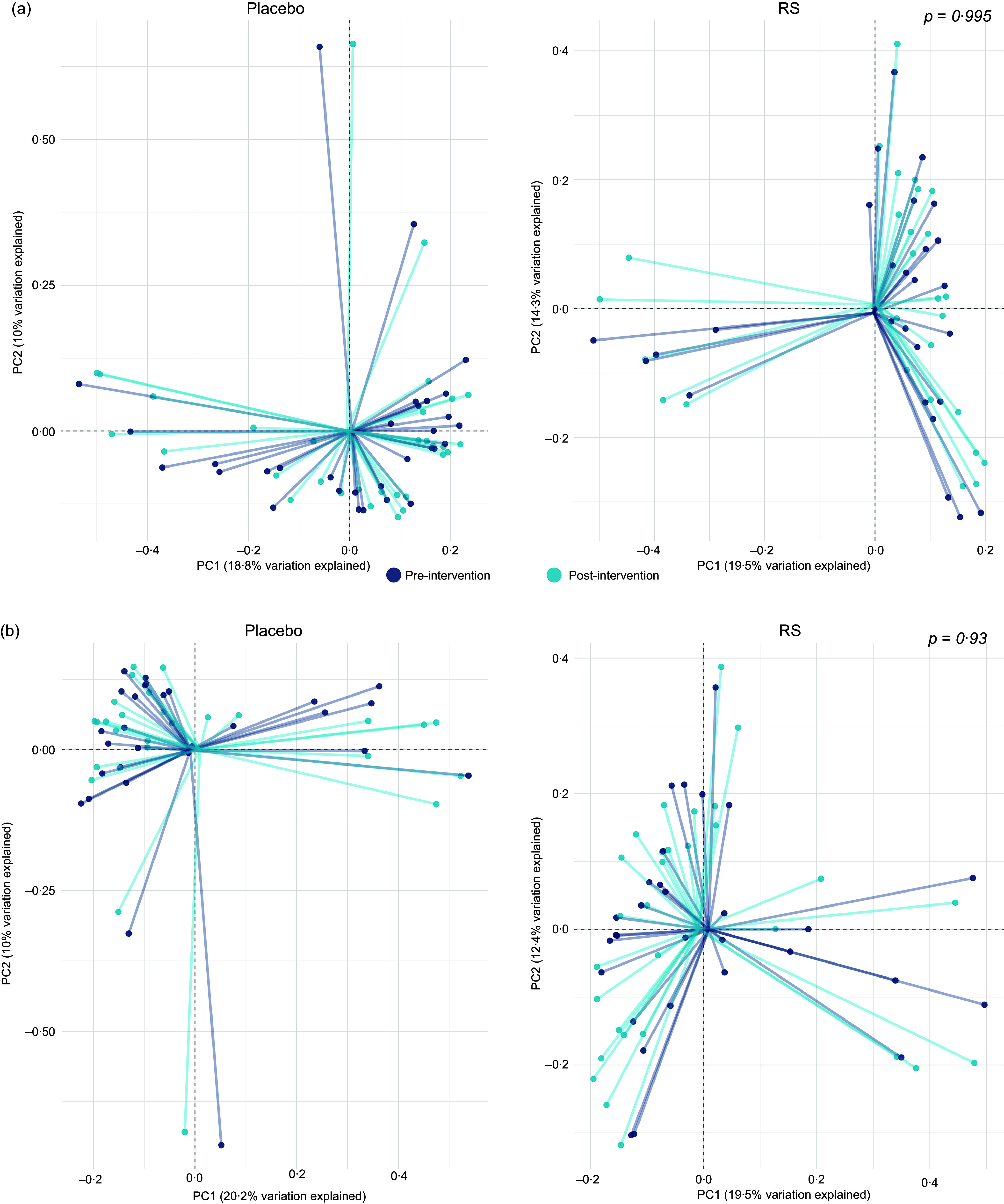
Principal coordinates analysis based on weighted Bray–Curtis distance metrics, illustrating microbial communities pre- and post-intervention in response to both interventions. (a) Impact of RS supplement intervention on microbial community composition. (b) Influence of PD supplement intervention on microbial community composition. PD, polydextrose; RS, resistant starch.

We further tested the effect of the intervention on beta-diversity metrics using both weighted and unweighted Bray–Curtis distances and found no effect of supplementation with RS (weighted *P* = 0·995, Fig. 3; unweighted: *P* = 1, online Supplementary Fig. 1) or PD (weighted: *P* = 0·93, Fig. 3; unweighted: *P =* 0·998, online Supplementary Fig. 1) compared with placebo.

### Effects of resistant starch and polydextrose on gut microbiota abundance

**Figure 4.**
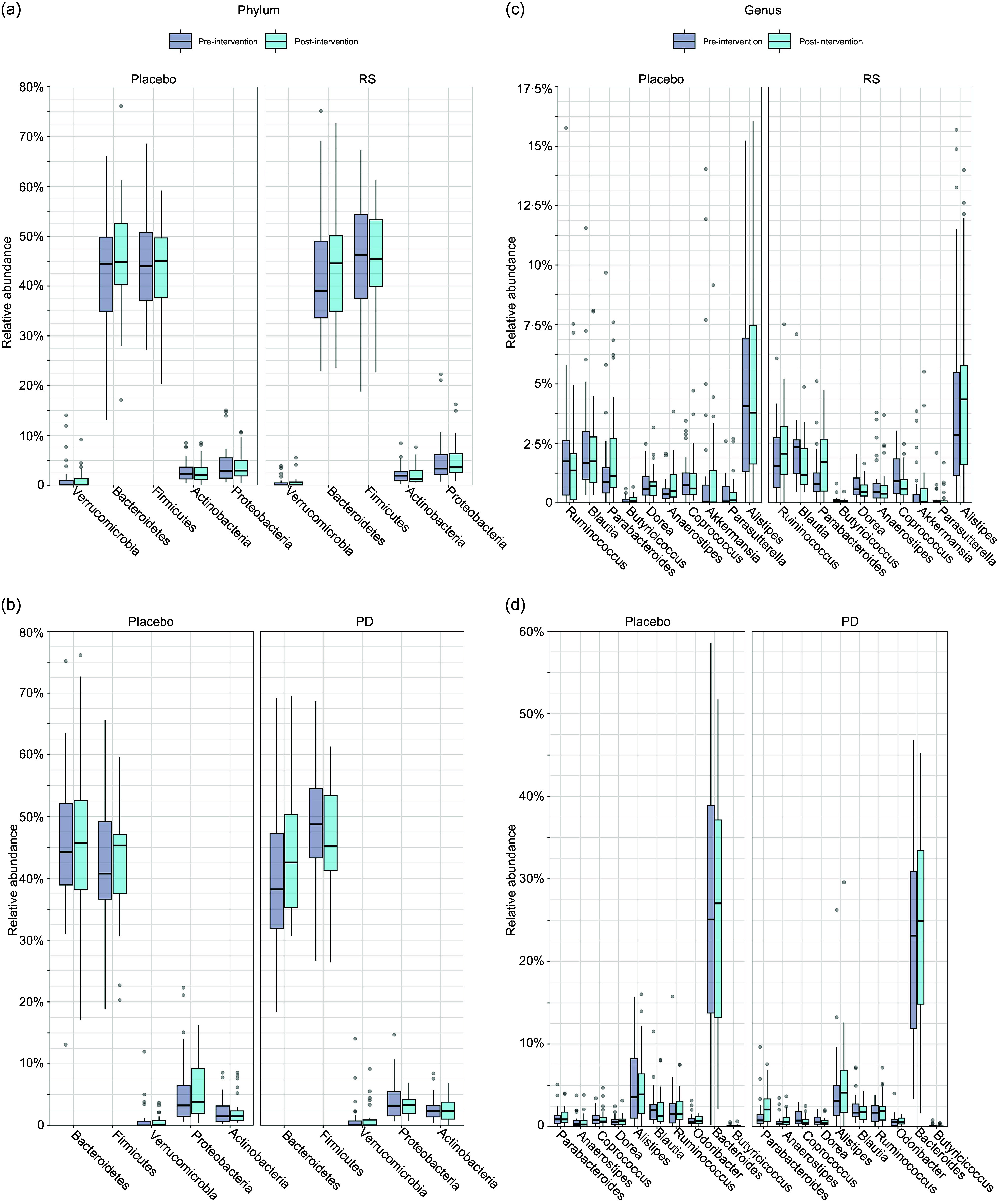
Effects of supplementation with RS (a and c) and PD (b and d) on the relative abundance of bacteria. Phyla and genera are ordered based on lowest *P* value. Boxes represent interquartile ranges, with lines denoting median. Abbreviations: PD, polydextrose; RS, resistant starch.

When investigating differences in relative abundance of bacteria at taxonomic levels, we detected no effects of either RS or PD compared with their respective placebos at the phylum or genus level (Fig. 4).

We further performed a sensitivity analysis to adjust for several confounders on the effects of dietary treatment on bacterial abundances, including, age, sex, BMI, endoscopy procedure, smoking status and baseline dietary factors (intakes of energy, fibre and alcohol). After adjusting for confounders and multiple testing (FDR > 0·05), we did not detect any effect of dietary supplements on microbiota abundances. Results were consistent when using the MaAsLin2 R package^(29)^ to conduct mixed effect modelling; no significant associations were identified after adjusting for participant ID as a random effect and the covariates listed above as fixed effects.

### Inter-individual response to intervention

Although no effects of the intervention on gut microbiota diversity were detected for the study participants as a whole, Fig. 2 and Fig. 3 show relatively large, inter-individual changes in diversity metrics. To identify participants who displayed consistent gut microbiota responses to the intervention, we performed DMM clustering. Microbiota profiles were clustered into three microbial ‘enterotypes’ (online Supplementary Fig. 2). Cluster 1 was composed mostly of *Bacteroides*, *Faecalibacterium* and unclassified Ruminococcaceae. Cluster 2 was driven by high relative abundances of *Bacteroides*, *Faecalibacterium* and unclassified Lachnospiraceae, while cluster 3 was driven by high relative abundance of *Prevotella*, *Faecalibacterium* and *Bacteroides* abundance.

**Figure 5.**
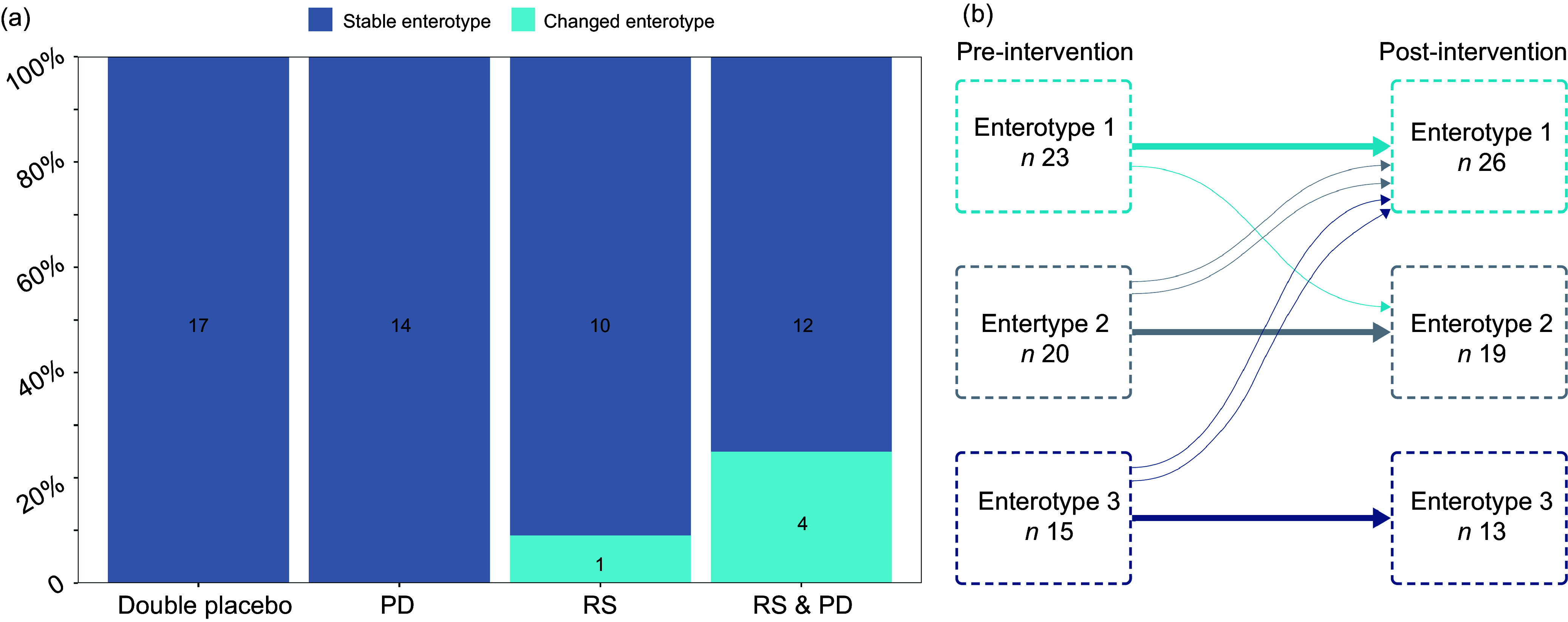
Dirichlet’s multinomial mixture clustering of participants. (a) Proportional bar plot summarising the number of participants whose enterotype was unchanged and those who changed gut microbial enterotype post-intervention. (b) Movement of participants between the identified clusters (enterotypes) between pre- and post-intervention. Thin lines represent individual participants. PD, polydextrose; RS, resistant starch.

In response to the intervention, most participants remained in their baseline enterotype (Fig. 5). However, five participants transitioned from one enterotype to another, four from the RS + PD arm and one participant from the RS arm (Fig. 5(a)). The participant from the RS arm that transitioned enterotypes went from enterotype 2 pre-intervention to enterotype 1 post-intervention. Two participants from the RS + PD group transitioned from enterotype 3 pre-intervention to enterotype 1 post-intervention; one participant went from enterotype 1 to enterotype 2 and one participant transitioned from enterotype 2 to enterotype 1 (Fig. 5(b)). The numbers switching enterotypes were not statistically significant between groups.

### Effects of resistant starch and polydextrose on metabolite concentrations in stool, plasma and urine

The results for the effects of RS and PD on fecal, plasma and urinary metabolite concentrations, including SCFA and branched-chain fatty acids (BCFA), are displayed in Table 2. Participants supplemented with PD had lower post-intervention fecal concentrations of isobutyrate (least square mean (sd) 1·28 (0·12) PD *v*. 1·67 (0·13) placebo, *P* = 0·042) and of valeric acid (least square mean (sd) 1·76 (0·17) PD *v*. 2·48 (0·17) placebo, *P* = 0·006). There were no effects of RS or PD, or an interaction between the two NDC, on the other fecal, plasma and urinary microbial metabolites measured.

**Table 2.** Effects of RS and PD on post-intervention microbial metabolite concentrations in stool, plasma and urine

Microbial metabolite	Effect of RS			Effect of PD			*P* interaction
	–	+	*P* value	–	+	*P* value	
Stool							
Acetate (µMol/ml)	41·5 (3·6)	50·1 (3·9)	0·112	48·6 (3·8)	43·0 (3·6)	0·300	0·306
Propionate (µMol/ml)	13·6 (1·4)	14·0 (1·5)	0·868	14·1 (1·5)	13·6 (1·4)	0·805	0·303
Butyrate (µMol/ml)	12·7 (1·4)	14·9 (1·5)	0·304	15·8 (1·5)	11·8 (1·4)	0·055	0·139
Isobutyrate (µMol/ml)	1·51 (0·12)	1·43 (0·13)	0·653	1·67 (0·13)	1·28 (0·12)	0·042*	0·264
2MB (µMol/ml)	0·96 (0·09)	0·84 (0·09)	0·378	1·00 (0·09)	0·80 (0·09)	0·115	0·488
Valeric acid (µMol/ml)	1·98 (0·17)	2·25 (0·18)	0·268	2·48 (0·18)	1·76 (0·17)	0·006*	0·880
Isovaleric acid (µMol/ml)	1·18 (0·11)	1·10 (0·11)	0·506	1·28 (0·11)	0·97 (0·11)	0·053	0·232
Lactic acid (µMol/ml)	1·14 (0·08)	1·09 (0·08)	0·721	1·09 (0·08)	1·14 (0·08)	0·719	0·176
Plasma							
Acetate (µMol/l)	103·1 (6·9)	101·1 (7·6)	0·848	100·9 (7·5)	103·4 (7·5)	0·810	0·895
Propionate (µMol/l)	7·9 (1·1)	5·8 (1·2)	0·202	6·7 (1·2)	7·0 (1·2)	0·842	0·192
Butyrate (µMol/l)	0·72 (0·18)	0·98 (0·19)	0·316	0·85 (0·19)	0·85 (0·19)	0·998	0·180
Isobutyrate (µMol/l)	2·37 (0·41)	2·27 (0·45)	0·877	2·20 (0·44)	2·43 (0·42)	0·710	0·463
2MB (µMol/l)	3·87 (0·71)	3·98 (0·78)	0·918	3·82 (0·78)	4·03 (0·74)	0·846	0·743
SCFA (µMol/l)	110·2 (7·5)	109·4 (8·2)	0·943	105·8 (8·1)	113·7 (7·7)	0·487	0·857
BCFA (µMol/l)	11·1 (2·0)	11·8 (2·2)	0·807	11·2 (2·1)	11·7 (2·0)	0·871	0·838
VFA (µMol/l)	121·4 (7·8)	121·2 (8·6)	0·988	117·3 (8·5)	125·3 (8·1)	0·508	0·904
Urine							
Acetate (µMol/l)	137·9 (29·7)	95·8 (32·1)	0·344	115·3 (32·1)	118·4 (30·3)	0·946	0·313
Propionate (µMol/l)	4·14 (0·49)	3·54 (0·52)	0·415	4·40 (0·52)	3·28 (0·49)	0·132	0·199
Butyrate (µMol/l)	3·03 (0·69)	2·33 (0·75)	0·501	3·48 (0·74)	1·88 (0·70)	0·129	0·185
Isobutyrate (µMol/l)	2·02 (0·30)	2·05 (0·33)	0·961	2·35 (0·32)	1·72 (0·31)	0·171	0·205
2MB (µMol/l)	2·69 (0·48)	2·63 (0·52)	0·930	2·71 (0·52)	2·61 (0·49)	0·893	0·345
SCFA (µMol/l)	144·9 (30·1)	101·8 (32·5)	0·338	123·1 (32·5)	123·7 (30·6)	0·990	0·299
BCFA (µMol/l)	6·15 (0·91)	5·89 (0·98)	0·844	6·58 (0·98)	5·45 (0·92)	0·410	0·197
VFA (µMol/l)	151·0 (30·1)	107·8 (32·6)	0·339	129·5 (32·6)	129·2 (30·7)	0·995	0·286

2MB, 2-methylbutyrate; BCFA, branched-chain fatty acids; PD, polydextrose; RS, resistant starch; SCFA, short-chain fatty acids; VFA, volatile fatty acids.

Data are presented as least squares means (LSM) for post-intervention data adjusted for pre-intervention measurement, age, sex, endoscopy procedure, BMI and smoking status (ANOVA GLM). Standard error of the mean (sem) are included in parentheses. *Significant effect of the intervention (*P* < 0·05). *P*_interaction_: *P* value for interaction effect of RS * PD.

When using delta change in metabolite concentrations between pre and post-intervention as the outcome, and after adjusting for age, sex, BMI, procedure type, smoking status, baseline dietary factors (energy, fibre and alcohol) and multiple testing, we noted that PD elevated fecal butyrate concentrations following the intervention (*β* (95 % CI) = 5·15 (0·55, 9·76), *P*_*adj*_ = 0·03).

We further investigated the correlations between metabolite concentrations derived from stool, plasma and urine samples pre- and post-intervention. We detected multiple significant intra-sample correlations between microbial metabolites; however, inter-sample correlations were scarcer (online Supplementary Fig. 3). Of note, there were relatively strong negative correlations between urinary acetate concentrations and fecal concentrations of isovaleric acid and 2-methyl-1-butanol at baseline (pre-intervention).

### Associations between gut microbiota and short-chain fatty acid concentrations

We further explored the relationship between changes in gut microbiota abundance and changes in fecal SCFA concentrations post-intervention. After adjusting for potential confounders, we detected possible associations between changes in gut microbial taxa, including *Hydrogenoanaerobacterium*, *Roseburia* and *Collinsella*, and changes in acetate (online Supplementary Fig. 4), propionate (online Supplementary Fig. 5), and butyrate concentrations (online Supplementary Fig. 6). However, none of these associations reached statistical significance following correction for multiple testing (FDR < 0·05).

## Discussion

To our knowledge, the DISC study is the first study to investigate the effects of both RS and PD, or their combination, on the diversity and composition of the gut microbiota, as well as on fecal, plasma and urinary metabolite concentrations, particularly SCFA, in healthy participants. Further, to our knowledge, this is the largest randomised controlled trial in which healthy participants have been supplemented with PD, and second largest which has supplemented with RS^(7)^ – the study by Alfa et al. included 84 participants^(30)^. The DISC study also had the longest duration of supplementation with PD^(7)^, and second longest to supplement with RS (the longest trial duration being 72 d^(30)^).

After adjusting for multiple testing and for potential confounders, we observed no effects of supplementation with RS and/or PD for 50 d on alpha-diversity, beta-diversity or on the relative abundance of bacteria phyla and genera. However, using clustering-based approaches that grouped individuals into enterotypes, we detected shifts in composition of the gut microbiota for some individuals in response to the RS and RS and PD intervention. This includes one participant supplemented with RS only and four participants supplemented with RS + PD (Fig. 5). Importantly, individuals randomised to the double placebo or PD arm did not change enterotype. This suggests that both the type and amount of dietary fibre may influence responses to intervention with larger amounts of dietary fibre (RS + PD) provoking enterotype change in more participants. The findings are in line with our earlier results on markers of colorectal cancer risk in the same cohort, where an effect of RS only on crypt cell proliferation was observed^(14)^.

In addition, the type of RS administered may be important in determining effects on the gut microbiome. Martinez et al. investigated the effects of supplementation with either 33 g per day of RS type 2 (a native granular starch consisting of ungelatinised granules) or type 4 (chemically modified starch) in ten healthy participants over 3 weeks and noted significant differences in their effects on human fecal microbiota composition^(31)^. The RS type 4 (given as Fibersym^®^) significantly decreased Firmicutes and increased Bacteroidetes and Actinobacteria, whereas RS type 2 (given as Hi-Maize 260) produced no changes at the phylum level^(31)^. In agreement with the findings from the present study, which used RS type 2, they found no effects of either type of RS on *α*-diversity^(31)^. In contrast, in a 3-months long randomised controlled trial in which eighty-two healthy participants were supplemented with a potato-derived RS type 2 or corn starch placebo, there were significant effects on two measures of *α*-diversity (Shannon diversity index and inverse Simpson index)^(30)^. RS type 2, used in the DISC study, is present in intact starch granules that can be found in foods such as bananas (4–5 g/100 g in ripe bananas and ∼18 g/100 g in green, unripe bananas^(32)^)^(33)^.

Only four other randomised controlled trials have explored the effects of supplementing healthy adults with PD on the gut microbiota^(34–37)^. Boler and colleagues examined the effects of supplementation with PD compared with soluble maize fibre or no fibre control on three specific fecal bacterial species and reported significant differences in post-intervention abundance of *Bifidobacterium* spp., *Lactobacillus* spp and *Escherichia coli* in those given PD^(35)^. In a placebo-controlled, double-blind, crossover study of thirty-one participants, 8 g of PD daily for 3 weeks significantly increased *α*-diversity (Simpson’s index) and the abundance of butyrate-producing *Ruminococcus intestinalis* and bacteria of the *Clostridium* clusters I, II and IV^(34)^. These changes were associated with lower fecal water genotoxicity, suggesting a reduction in risk factors that may be associated with early stages of colorectal carcinogenesis^(34)^. There were no effects of supplementation with PD on fecal SCFA concentrations^(34)^. Beards and colleagues supplemented forty healthy participants with chocolate products containing different blends of sucrose replacers (RS, PD or maltitol) for 6 weeks and found a significant increase in numbers of *lactobacilli*, *Clostridium histolyticum/perfringens* populations, Bacteroides, *E. rectale*, *R. flavefaciens* and total bacteria^(36)^. Over the 6-week period, there was also a significant increase in fecal acetate, propionate and butyrate concentrations^(36)^. In healthy Chinese adults, supplementation with PD for 28 d increased *Lactobacillus* and *Bifidobacterium* species and decreased *Bacteroides* species^(37)^. Higher doses of PD increased fecal concentrations of acetate, butyrate and isobutyrate, and, perhaps surprisingly, resulted in higher colonocyte proliferation^(37)^. In contrast, in twenty-one healthy adult men with a low habitual dietary fibre intake (∼13–15 g/d), supplementation with 21 g/d of PD for 21 d resulted in lower fecal acetate, propionate and butyrate concentrations compared with soluble maize fibre or no fibre control^(35)^. In agreement with the findings of Boler and colleagues^(35)^, in the DISC study, lower fecal concentrations of butyrate and valeric acid SCFA and of the BCFA isobutyrate were observed in participants supplemented with PD compared with placebo. We did not observe significant effects of RS, compared with placebo, on fecal, urinary or plasma SCFA. Although there is evidence that RS is one of the most effective dietary fibres at modulating the gut microbiota to stimulate butyrate production^(38)^, our systematic dose–response studies in the rat model have shown that the extent to which SCFA patterns are driven towards high molar proportion of butyrate depends on RS dose with lower molar proportions of butyrate observed with higher RS doses^(39)^. Importantly, in humans, the effects of RS on large bowel SCFA patterns vary at the individual participant level,^(40,41)^ and some studies have reported no effects on butyrate concentrations^(42)^. In addition, the effects of RS on butyrate, and other SCFA, are influenced by RS type^(43)^. For example, although potato dish supplementation (providing RS type 2 and/or 3) for 4 weeks increased the abundance of butyrate-producing *Roseburia faecis* in healthy adults, there were no significant effects on fecal SCFA concentrations^(44)^. When healthy young adults were supplemented with different types of RS for 2 weeks, only that from potatoes, but not RS from maize, increased fecal butyrate concentrations^(41)^.

The lack of consistency in observations between the present study and some other studies of RS and PD supplementation in humans reflects the heterogeneity in findings reported in the systematic review and meta-analysis of effects of dietary fibre on the gut microbiome by So and colleagues^(7)^. In addition to heterogeneity in characteristics of the study participants, this lack of consistency in response to supplementation may be explained by several factors related to the protocols for the intervention studies with the most prominent differences being type and dose of dietary fibre, study duration, comparator treatment and study design (most studies to date have been crossover trials)^(45)^. For example, RS type 4 may lead to larger changes in the composition of the gut microbiota than other RS types^(31)^. Between study differences in findings may also be due to differences in the methods used to analyse the gut microbiota and to quantify concentrations of SCFA and other microbial metabolites.

### Strengths and limitations

A strength of this study is that it was an randomised controlled trial that investigated the effects of equal doses of two contrasting types of dietary fibres (RS and PD) given for 50 d, which is one of the largest and longest duration studies of the effects of dietary fibre on the gut microbiome^(7)^. The doses used (12 g of PD and 23 g RS per day) were designed to provide similar quantities of additional dietary fibre i.e. 11–12 g per day. As adults (aged 19–64 years) in the UK are estimated to consume, on average, 19·7 g of dietary fibre (AOAC definition) per day^(46)^, the doses given would enhance fibre intake to the recommended level i.e. 30 g/d^(47)^. Further, the ingredients were chosen as intervention agents because they can be incorporated into foods conveniently to enhance dietary fibre intake. Although RS and PD did not change the composition of the gut microbiota in this study, dietary fibre has a range of other beneficial effects including improving gut motility and contributing to metabolic health. Gastrointestinal transit time is a major modulator of the gut microbiome and of microbial metabolites both *in vitro* ^(48)^ and *in vivo* ^(49)^. Whilst some dietary fibres may impact gastrointestinal transit time^(50–52)^, there is much less evidence for a consistent effect of RS on transit time^(39,53–55)^. Since we did not observe significant effects of RS or PD on the measured outcomes, we do not anticipate any effects on transit time.

We also performed sensitivity analyses to adjust for several potential confounders i.e. age, sex, endoscopy procedure at baseline, baseline dietary fibre intake and BMI. The results from these sensitivity analyses were comparable to those from our initial analyses, confirming our findings.

Another strength is that, in addition to characterising changes in the gut microbiota, we investigated effects on SCFA and other microbial metabolites in three different sample types i.e. stool, plasma and urine. However, fecal concentrations of SCFA may not be fully representative of intraluminal SCFA. For example, autopsy study of sudden death victims showed that concentrations of SCFA fell from the proximal to the distal colon^(56)^. However, concentration of total SCFA in faeces was similar to those in the sigmoid and rectum^(56)^.

A limitation of our study is that it may have been underpowered given that *a priori* sample size calculations were not conducted and that this was a secondary analysis using available data and samples. Although the DISC study included seventy-five participants, for the current investigation, paired baseline and follow-up data were available for a subset of fifty-eight participants only which limited our statistical power to detect effects of the intervention. That said, this is one of the largest randomised controlled studies on the effects of RS and PD on gastrointestinal microbiota and their metabolites in humans. Further, given the considerable inter-individual variation observed both at baseline and in response to the dietary fibre supplementation, the study duration and size may not have been sufficient to detect intervention effects at a group level.

Another limitation of this study is that dietary data were self-reported, which can introduce self-report bias^(57)^. The mean habitual dietary fibre intake at baseline in our study was 22·5 g per day (mean intake by adults in the UK is approximately 18 g/d), which may suggest recruitment of participants with relatively healthier diets, despite the majority of participants having obesity, or an over-estimation of dietary fibre intake. We asked participants to maintain their habitual diet, but changes in other lifestyle factors such as physical activity, with potential to influence the gut microbiota and/or their metabolism, may have occurred during the intervention period. Lastly, all fifty-eight participants in this study identified as White British, restricting the generalisability of our findings to wider, more ethnically diverse populations.

### Conclusions

In the DISC study, supplementation with RS and/or PD for 50 d did not elicit changes in the gut microbiota of healthy adults. Larger and longer duration studies that supplement with higher doses of dietary fibre are required to further investigate the effects of dietary fibre on the gut microbiome and associated metabolites. Such studies should focus on the effects of different fibre subtypes (e.g. RS type 1 *v*. RS type 2), be conducted in participants with different health status (e.g. individuals with bacterial dysbiosis or patients with type 2 diabetes^(58)^ may be more responsive to dietary intervention) and use alternate study designs, including repeated measures and cross-over studies.

## Supporting information

Malcomson et al. supplementary material Malcomson et al. supplementary material

## Supplementary material

For supplementary material/s referred to in this article, please visit https://doi.org/10.1017/S000711452400271X

## References

[1] McKeown NM , Fahey GC Jr , Slavin J , et al. (2022) Fibre intake for optimal health: how can healthcare professionals support people to reach dietary recommendations? BMJ 378, e054370. 35858693 10.1136/bmj-2020-054370 PMC9298262

[2] Reynolds A , Mann J , Cummings J , et al. (2019) Carbohydrate quality and human health: a series of systematic reviews and meta-analyses. Lancet 393, 434–445. 30638909 10.1016/S0140-6736(18)31809-9

[3] Cummings JH & Stephen AM (2007) Carbohydrate terminology and classification. Eur J Clin Nutr 61, S5–18. 17992187 10.1038/sj.ejcn.1602936

[4] Stowell JD (2009) Prebiotic potential of polydextrose. In Prebiotics and Probiotics Science and Technology, p. 337 [ DC Rastall and GR Gibson , editors]. New York: Springer Science + Business Media, LLC.

[5] Raigond P , Dutt S & Singh B (2019) Resistant starch in food. In Bioactive Molecules in Food, pp. 815–846 (J-M Mérillon and KG Ramawat , editors). Cham: Springer International Publishing.

[6] Swanson KS , Gibson GR , Hutkins R , et al. (2020) The International Scientific Association for Probiotics and Prebiotics (ISAPP) consensus statement on the definition and scope of synbiotics. Nat Rev Gastroenterol Hepatol 17, 687–701. 32826966 10.1038/s41575-020-0344-2 PMC7581511

[7] So D , Whelan K , Rossi M , et al. (2018) Dietary fiber intervention on gut microbiota composition in healthy adults: a systematic review and meta-analysis. Am J Clin Nutr 107, 965–983. 29757343 10.1093/ajcn/nqy041

[8] Canani RB , Costanzo MD , Leone L , et al. (2011) Potential beneficial effects of butyrate in intestinal and extraintestinal diseases. World J Gastroenterol 17, 1519–1528. 21472114 10.3748/wjg.v17.i12.1519 PMC3070119

[9] Chambers ES , Byrne CS , Morrison DJ , et al. (2019) Dietary supplementation with inulin-propionate ester or inulin improves insulin sensitivity in adults with overweight and obesity with distinct effects on the gut microbiota, plasma metabolome and systemic inflammatory responses: a randomised cross-over trial. Gut 68, 1430–1438. 30971437 10.1136/gutjnl-2019-318424 PMC6691855

[10] Chambers ES , Viardot A , Psichas A , et al. (2015) Effects of targeted delivery of propionate to the human colon on appetite regulation, body weight maintenance and adiposity in overweight adults. Gut 64, 1744–1754. 25500202 10.1136/gutjnl-2014-307913 PMC4680171

[11] Rios-Covian D , Gonzalez S , Nogacka AM , et al. (2020) An overview on fecal branched short-chain fatty acids along human life and as related with body mass index: associated dietary and anthropometric factors. Front Microbiol 11, 973. 32547507 10.3389/fmicb.2020.00973 PMC7271748

[12] Boler BM , Serao MC , Bauer LL , et al. (2011) Digestive physiological outcomes related to polydextrose and soluble maize fibre consumption by healthy adult men. Br J Nutr 106, 1864–1871. 21736814 10.1017/S0007114511002388

[13] Malcomson FC , Willis ND , McCallum I , et al. (2017) Effects of supplementation with nondigestible carbohydrates on fecal calprotectin and on epigenetic regulation of SFRP1 expression in the large-bowel mucosa of healthy individuals. Am J Clin Nutr 105, 400–410. 28077379 10.3945/ajcn.116.135657 PMC5267298

[14] Malcomson FC , Willis ND , McCallum I , et al. (2020) Resistant starch supplementation increases crypt cell proliferative state in the rectal mucosa of older healthy participants. Br J Nutr 124, 374–385. 32279690 10.1017/S0007114520001312 PMC7369377

[15] Malcomson FC , Willis ND , McCallum I , et al. (2017) Non-digestible carbohydrates supplementation increases miR-32 expression in the healthy human colorectal epithelium: a randomized controlled trial. Mol Carcinog 56, 2104–2111. 28418082 10.1002/mc.22666 PMC5573932

[16] Kroke A , Klipstein-Grobusch K , Voss S , et al. (1999) Validation of a self-administered food-frequency questionnaire administered in the European Prospective Investigation into Cancer and Nutrition (EPIC) Study: comparison of energy, protein, and macronutrient intakes estimated with the doubly labeled water, urinary nitrogen, and repeated 24-h dietary recall methods. Am J Clin Nutr 70, 439–447. 10500011 10.1093/ajcn/70.4.439

[17] Kozich JJ , Westcott SL , Baxter NT , et al. (2013) Development of a dual-index sequencing strategy and curation pipeline for analyzing amplicon sequence data on the MiSeq Illumina sequencing platform. Appl Environ Microbiol 79, 5112–5120. 23793624 10.1128/AEM.01043-13 PMC3753973

[18] Rognes T , Flouri T , Nichols B , et al. (2016) VSEARCH: a versatile open source tool for metagenomics. PeerJ 4, e2584. 27781170 10.7717/peerj.2584 PMC5075697

[19] Peuranen S , Tiihonen K , Apajalahti J , et al. (2004) Combination of polydextrose and lactitol affects microbial ecosystem and immune responses in rat gastrointestinal tract. Br J Nutr 91, 905–914. 15182394 10.1079/BJN20041114

[20] Morrison DJ , Cooper K , Waldron S , et al. (2004) A streamlined approach to the analysis of volatile fatty acids and its application to the measurement of whole-body flux. Rapid Commun Mass Spectrom 18, 2593–2600. 15468137 10.1002/rcm.1662

[21] Dronamraju SS , Coxhead JM , Kelly SB , et al. (2009) Cell kinetics and gene expression changes in colorectal cancer patients given resistant starch: a randomised controlled trial. Gut 58, 413–420. 18978177 10.1136/gut.2008.162933

[22] R Core Team (2020) R: A Language and Environment for Statistical Computing, 4.3.1 ed. Vienna, Austria: R Foundation for Statistical Computing.

[23] Alkek Center for Metagenomics and Microbiome Research (2019) ATIMA (Agile toolKit for Inclusive Microbial Analyses). https://atima.research.bcm.edu/ (accessed September 2023).

[24] Benjamini Y & Hochberg Y (1995) Controlling the false discovery rate: a practical and powerful approach to multiple testing. J Royal Stat Soc Ser B (Methodological) 57, 289–300.

[25] Holmes I , Harris K & Quince C (2012) Dirichlet multinomial mixtures: generative models for microbial metagenomics. Plos One 7, e30126. 22319561 10.1371/journal.pone.0030126 PMC3272020

[26] Bliss CI , Greenwood ML & White ES (1956) A rankit analysis of paired comparisons for measuring the effect of sprays on flavor. Biom 12, 381–403.

[27] Englyst HN , Quigley ME & Hudson GJ (1994) Determination of dietary fibre as non-starch polysaccharides with gas-liquid chromatographic, high-performance liquid chromatographic or spectrophotometric measurement of constituent sugars. Analyst 119, 1497–1509. 7943740 10.1039/an9941901497

[28] King CH , Desai H , Sylvetsky AC , et al. (2019) Baseline human gut microbiota profile in healthy people and standard reporting template. PLOS ONE 14, e0206484. 31509535 10.1371/journal.pone.0206484 PMC6738582

[29] Mallick H , Rahnavard A , McIver LJ , et al. (2021) Multivariable association discovery in population-scale meta-omics studies. PLoS Comput Biol 17, e1009442. 34784344 10.1371/journal.pcbi.1009442 PMC8714082

[30] Alfa MJ , Strang D , Tappia PS , et al. (2018) A randomized trial to determine the impact of a digestion resistant starch composition on the gut microbiome in older and mid-age adults. Clin Nutr 37, 797–807. 28410921 10.1016/j.clnu.2017.03.025

[31] Martínez I , Kim J , Duffy PR , et al. (2010) Resistant starches types 2 and 4 have differential effects on the composition of the fecal microbiota in human subjects. Plos One 5, e15046. 21151493 10.1371/journal.pone.0015046 PMC2993935

[32] Phillips KM , McGinty RC , Couture G , et al. (2021) Dietary fiber, starch, and sugars in bananas at different stages of ripeness in the retail market. Plos One 16, e0253366. 34237070 10.1371/journal.pone.0253366 PMC8266066

[33] Mathers JC (2023) Dietary fibre and health: the story so far. Proc Nutr Soc 82, 120–129. 36786062 10.1017/S0029665123002215

[34] Costabile A , Fava F , Röytiö H , et al. (2012) Impact of polydextrose on the faecal microbiota: a double-blind, crossover, placebo-controlled feeding study in healthy human subjects. Br J Nutr 108, 471–481. 22099384 10.1017/S0007114511005782

[35] Boler BMV , Serao MCR , Bauer LL , et al. (2011) Digestive physiological outcomes related to polydextrose and soluble maize fibre consumption by healthy adult men. Br J Nutr 106, 1864–1871. 21736814 10.1017/S0007114511002388

[36] Beards E , Tuohy K & Gibson G (2010) A human volunteer study to assess the impact of confectionery sweeteners on the gut microbiota composition. Br J Nutr 104, 701–708. 20370946 10.1017/S0007114510001078

[37] Jie Z , Bang-Yao L , Ming-Jie X , et al. (2000) Studies on the effects of polydextrose intake on physiologic functions in Chinese people. Am J Clin Nutr 72, 1503–1509. 11101478 10.1093/ajcn/72.6.1503

[38] Cummings JH , Macfarlane GT & Englyst HN (2001) Prebiotic digestion and fermentation. Am J Clin Nutr 73, 415S–420S. 11157351 10.1093/ajcn/73.2.415s

[39] Mathers JC , Smith H & Carter S (1997) Dose-response effects of raw potato starch on small-intestinal escape, large-bowel fermentation and gut transit time in the rat. Br J Nutr 78, 1015–1029. 9497449

[40] Venkataraman A , Sieber JR , Schmidt AW , et al. (2016) Variable responses of human microbiomes to dietary supplementation with resistant starch. Microbiome 4, 33. 27357127 10.1186/s40168-016-0178-x PMC4928258

[41] Baxter NT , Schmidt AW , Venkataraman A , et al. (2019) Dynamics of human gut microbiota and short-chain fatty acids in response to dietary interventions with three fermentable fibers. mBio 10, 10–1128. 10.1128/mBio.02566-18 PMC6355990 30696735

[42] DeMartino P & Cockburn DW (2020) Resistant starch: impact on the gut microbiome and health. Curr Opin Biotechnol 61, 66–71. 31765963 10.1016/j.copbio.2019.10.008

[43] Teichmann J & Cockburn DW (2021) *In vitro* fermentation reveals changes in butyrate production dependent on resistant starch source and microbiome composition. Front Microbiol 12, 640253. 33995299 10.3389/fmicb.2021.640253 PMC8117019

[44] DeMartino P , Johnston EA , Petersen KS , et al. (2022) Additional resistant starch from one potato side dish per day alters the gut microbiota but not fecal short-chain fatty acid concentrations. Nutrients 14, 721. 35277080 10.3390/nu14030721 PMC8840755

[45] Dobranowski PA & Stintzi A (2021) Resistant starch, microbiome, and precision modulation. Gut Microbes 13, 1926842. 34275431 10.1080/19490976.2021.1926842 PMC8288039

[46] PHE/FSA (2020) National Diet and Nutrition Survey Rolling Programme Years 9 to 11 (2016/2017 to 2018/2019). A Survey Carried Out on Behalf of Public Health England and the Food Standards Agency. https://assets.publishing.service.gov.uk/media/5fd23324e90e07662b09d91a/NDNS_UK_Y9–11_report.pdf (accessed October 2023).

[47] SACN (2015) SACN Carbohydrates and Health Report. London. TSO. https://www.gov.uk/government/publications/sacn-carbohydrates-and-health-report (accessed October 2023)

[48] Minnebo Y , Delbaere K , Goethals V , et al. (2023) Gut microbiota response to *i*n vitro transit time variation is mediated by microbial growth rates, nutrient use efficiency and adaptation to *i*n vivo transit time. Microbiome 11, 240. 37926855 10.1186/s40168-023-01691-y PMC10626715

[49] Asnicar F , Leeming ER , Dimidi E , et al. (2021) Blue poo: impact of gut transit time on the gut microbiome using a novel marker. Gut 70, 1665. 33722860 10.1136/gutjnl-2020-323877 PMC8349893

[50] Muller-Lissner SA (1988) Effect of wheat bran on weight of stool and gastrointestinal transit time: a meta analysis. Br Med J (Clin Res Ed) 296, 615–617. 10.1136/bmj.296.6622.615 PMC2545244 2832033

[51] Payler DK , Pomare EW , Heaton KW , et al. (1975) The effect of wheat bran on intestinal transit. Gut 16, 209–213. 1091532 10.1136/gut.16.3.209 PMC1410956

[52] Muir JG , Yeow EG , Keogh J , et al. (2004) Combining wheat bran with resistant starch has more beneficial effects on fecal indexes than does wheat bran alone. Am J Clin Nutr 79, 1020–1028. 15159232 10.1093/ajcn/79.6.1020

[53] Cummings JH , Beatty ER , Kingman SM , et al. (1996) Digestion and physiological properties of resistant starch in the human large bowel. Br J Nutr 75, 733–747. 8695600 10.1079/bjn19960177

[54] Tomlin J & Read NW (1990) The effect of resistant starch on colon function in humans. Br J Nutr 64, 589–595. 1699597 10.1079/bjn19900058

[55] Sharma A , Yadav BS & Yadav RB (2008) Resistant starch: physiological roles and food applications. Food Rev Int 24, 193–234.

[56] Cummings JH , Pomare EW , Branch WJ , et al. (1987) Short chain fatty acids in human large intestine, portal, hepatic and venous blood. Gut 28, 1221–1227. 3678950 10.1136/gut.28.10.1221 PMC1433442

[57] Penn L , Boeing H , Boushey CJ , et al. (2010) Assessment of dietary intake: nuGO symposium report. Genes Nutr 5, 205–213. 10.1007/s12263-010-0175-9 PMC2935535 21052527

[58] Ojo O , Ojo OO , Zand N , et al. (2021) The effect of dietary fibre on gut microbiota, lipid profile, and inflammatory markers in patients with type 2 diabetes: a systematic review and meta-analysis of randomised controlled trials. Nutrients 13, 1805. 34073366 10.3390/nu13061805 PMC8228854

