# Supplementary material for: Effects of non-digestible carbohydrates on gut microbiota and microbial metabolites: a randomised, controlled dietary intervention in healthy individuals
Source: Br J Nutr. Author manuscript; Available in PMC 2024 Nov 13. (PMC7616798; doi:10.1017/S000711452400271X)
Supplement: Supplementary file 1 [file EMS199722-supplement-Supplementary_Material.pdf]

Supplementary Material

DISC Study Methods

Potential study participants were identified using endoscopy patient lists and sent a study invitation letter with detailed information about the DISC Study at least five days prior to their hospital appointment. At endoscopy, potential study participants were screened for exclusion criteria.

Exclusion criteria included: aged<16 or >85 years, pregnant or planning to become pregnant, diabetes mellitus, Familial adenomatous polyposis syndrome, Lynch Syndrome, known colorectal tumour or prior CRC, prior colorectal resection, active colonic inflammation at endoscopy, Iatrogenic perforation at endoscopy, Incomplete left-sided examination, Colorectal carcinoma discovered at endoscopy or histology, chemotherapy in the last six months, administering non-steroidal anti-inflammatories (NSAIDs) e.g. aspirin, anti-coagulants e.g. warfarin or immunosuppressive medication e.g. methotrexate.

At least one week post endoscopy, participants were randomised to one of four dietary interventions by selecting a sealed, opaque envelope labelled A, B, C or D. Participant randomisation was stratified by pre-intervention endoscopy procedure to avoid effects of the different bowel preparations.

Participants were randomised to one of four groups:

- Double placebo: 12g Maltodextrin and 23g Amioca starch
- RS: 23g Hi-maize® 260 and 12g Maltodextrin
- PD: 12g Litesse® Ultra™ and 23g Amioca starch
- Double intervention: 23g Hi-maize® 260 and 12g Litesse® Ultra™

RS was provided in the form of Hi-maize® 260 (Ingredion™, Food Innovation, USA), a type II RS isolated from high-amylose corn hybrids. As approximately 60% of Hi-maize® 260 is RS, participants took 23g of Hi-maize® 260 or placebo to yield a similar amount of active agent as those in the PD group. PD was supplied in the form of Litesse® Ultra™ (International Flavors & Fragances™ Danisco®, Finland), a glucose polymer produced from corn-derived sorbitol, dextrose and citric acid. Participants supplemented with PD were given 12g of Litesse® Ultra™ per day. The placebos for

RS and PD were Amioca starch and Maltodextrin respectively. Both are easily digested and absorbed in the small intestine and therefore have no effects on the large bowel.

The intervention supplementation period was 50 days. The intervention agents were supplied in a white powdered form in foil sachets packed into boxes containing a week's worth of sachets. Participants consumed 35g of intervention supplement per day divided into four sachets (two sachets of each intervention agent). The powdered supplements were consumed by adding to cold food or liquids such as yoghurt, orange juice or water. Participants were asked to retain all their sachets, including those that were not consumed. To measure compliance, the number of consumed and not consumed sachets were counted for each participant.

Supplementary Table 1: Pre- and post-intervention microbial metabolite concentrations in stool, plasma, and urine according to RS and PD allocation

Microbial metabolite	RS allocation				PD allocation			
	-		+		-		+	
Stool	**Pre**	**Post**	**Pre**	**Post**	**Pre**	**Post**	**Pre**	**Post**
**Acetate (µMol/mL)**	41.0 (2.8)	39.6 (3.7)	49.3 (3.7)	51.2 (4.0)	44.2 (3.2)	47.0 (4.3)	45.5 (3.4)	43.1 (3.6)
**Propionate (µMol/mL)**	13.4 (1.0)	13.0 (1.6)	14.7 (1.4)	14.3 (1.6)	13.2 (1.1)	13.6 (1.5)	14.7 (1.3)	13.6 (1.8)
**Butyrate (µMol/mL)**	11.8 (1.2)	11.7 (1.4)	15.6 (1.7)	15.4 (1.9)	12.5 (1.4)	14.8 (2.0)	14.5 (1.5)	12.1 (1.3)
**Isobutyrate (µMol/mL)**	1.9 (0.1)	1.6 (0.1)	1.7 (0.2)	1.4 (0.1)	1.7 (0.1)	1.7 (0.1)	1.9 (0.2)	1.3 (0.1)
**2MB (µMol/mL)**	1.2 (0.1)	1.0 (0.1)	1.0 (0.1)	0.8 (0.1)	1.1 (0.1)	1.0 (0.1)	1.2 (0.1)	0.8 (0.1)
**Valeric acid (µMol/mL)**	2.1 (0.2)	2.0 (0.2)	2.4 (0.2)	2.2 (0.2)	2.1 (0.2)	2.5 (0.2)	2.4 (0.2)	1.8 (0.2)
**Isovaleric acid (µMol/mL)**	1.5 (0.1)	1.2 (0.1)	1.2 (0.1)	1.0 (0.1)	1.3 (0.1)	1.3 (0.1)	1.5 (0.2)	1.0 (0.1)
**Lactic acid (µMol/mL)**	1.3 (0.2)	1.0 (0.1)	1.1 (0.1)	1.1 (0.0)	1.1 (0.1)	1.1 (0.0)	1.1 (0.1)	1.1 (0.1)
Plasma								
**Acetate (µMol/L)**	103.5 (9.2)	103.8 (6.7)	100.7 (8.5)	98.6 (8.9)	98.4 (10.5)	100.4 (7.2)	105.9 (7.2)	102.3 (8.1)
**Propionate (µMol/L)**	3.5 (0.5)	5.3 (1.6)	6.5 (2.8)	8.8 (4.8)	3.1 (0.4)	3.7 (0.3)	6.5 (2.5)	10.0 (4.5)
**Butyrate (µMol/L)**	0.5 (0.1)	0.7 (0.1)	0.5 (0.1)	1.0 (0.4)	0.4 (0.1)	0.5 (0.1)	0.7 (0.2)	1.2 (0.4)
**Isobutyrate (µMol/L)**	2.1 (0.3)	2.4 (0.4)	1.9 (0.4)	2.2 (0.4)	1.8 (0.3)	2.2 (0.5)	2.2 (0.4)	2.4 (0.4)
**2MB (µMol/L)**	3.2 (0.5)	3.8 (0.7)	3.5 (0.6)	4.0 (0.8)	2.8 (0.6)	3.6 (0.9)	3.9 (0.5)	4.2 (0.7)
**SCFA (µMol/L)**	107.4 (9.4)	109.8 (6.8)	107.8 (8.8)	108.4 (9.6)	101.9 (10.8)	104.5 (7.4)	113.0 (7.2)	113.5 (8.6)
**BCFA (µMol/L)**	10.1 (1.5)	11.3 (2.0)	9.9 (1.7)	11.5 (2.3)	8.9 (1.6)	10.9 (2.5)	11.1 (1.5)	11.8 (1.8)
**VFA (µMol/L)**	117.5 (10.2)	121.1 (7.6)	117.7 (9.6)	119.9 (10.5)	110.8 (11.8)	115.4 (8.8)	124.1 (7.7)	125.3 (9.1)
Urinary								
**Acetate (µMol/L)**	115.3 (23.5)	134.9 (37.0)	846 (614)	92.1 (17.7)	111.1 (20.7)	106.9 (0.8)	777 (553)	1.6 (0.5)
**Propionate (µMol/L)**	3.9 (0.5)	4.1 (0.5)	8.8 (4.2)	3.4 (0.5)	4.2 (0.6)	4.2 (0.6)	7.9 (3.8)	3.4 (0.4)
**Butyrate (µMol/L)**	2.2 (0.5)	3.0 (0.8)	6.4 (4.1)	2.1 (0.6)	2.8 (0.9)	3.3 (0.9)	5.4 (3.6)	1.9 (0.5)
**Isobutyrate (µMol/L)**	1.9 (0.4)	2.0 (0.3)	5.8 (3.5)	2.0 (0.3)	1.8 (0.4)	2.1 (0.4)	5.5 (3.1)	1.9 (0.3)
**2MB (µMol/L)**	3.4 (0.9)	2.6 (0.5)	4.9 (2.1)	2.6 (0.6)	2.8 (0.9)	2.3 (0.5)	5.4 (1.9)	2.8 (0.6)
**SCFA (µMol/L)**	121.4 (23.5)	141.9 (37.5)	861 (622)	97.6 (17.9)	118.1 (20.6)	114.4 (21.8)	790 (561)	127.7 (37.1)
**BCFA (µMol/L)**	6.8 (1.5)	5.9 (1.0)	12.3 (6.4)	5.8 (1.1)	6.1 (1.5)	5.8 (1.0)	13.3 (5.8)	6.0 (1.1)
**VFA (µMol/L)**	128.3 (23.7)	147.8 (37.5)	874 (629)	103.4 (18.3)	124.1 (20.8)	120.2 (22.2)	803 (566)	133.7 (37.0)

Data are presented as means and standard error of the mean (SEM) in parentheses.
Abbreviations: 2MB, 2-methylbutyrate; BCFA, branched-chain fatty acids; PD, polydextrose; RS, resistant starch; SCFA, short-chain fatty acids; VFA, volatile fatty acids.

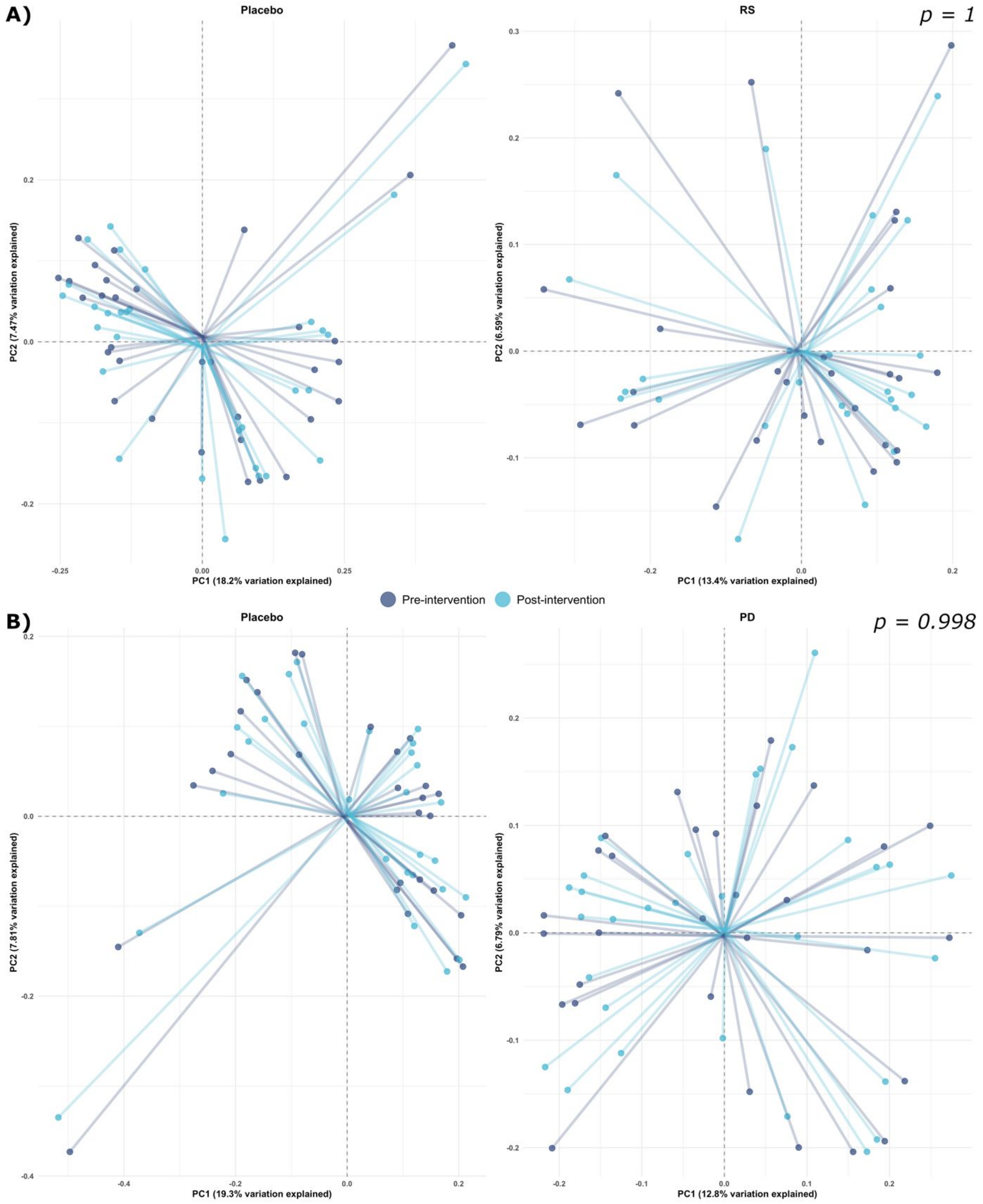

Supplementary Figure 1. Principal Coordinates Analysis based on unweighted Bray-Curtis distance metrics, illustrating microbial communities pre- and post-intervention in response to both interventions. A) Impact of RS intervention on microbial community composition. **B)** Impact of PD intervention on microbial

community composition.
Abbreviations: RS, resistant starch; PD, polydextrose.

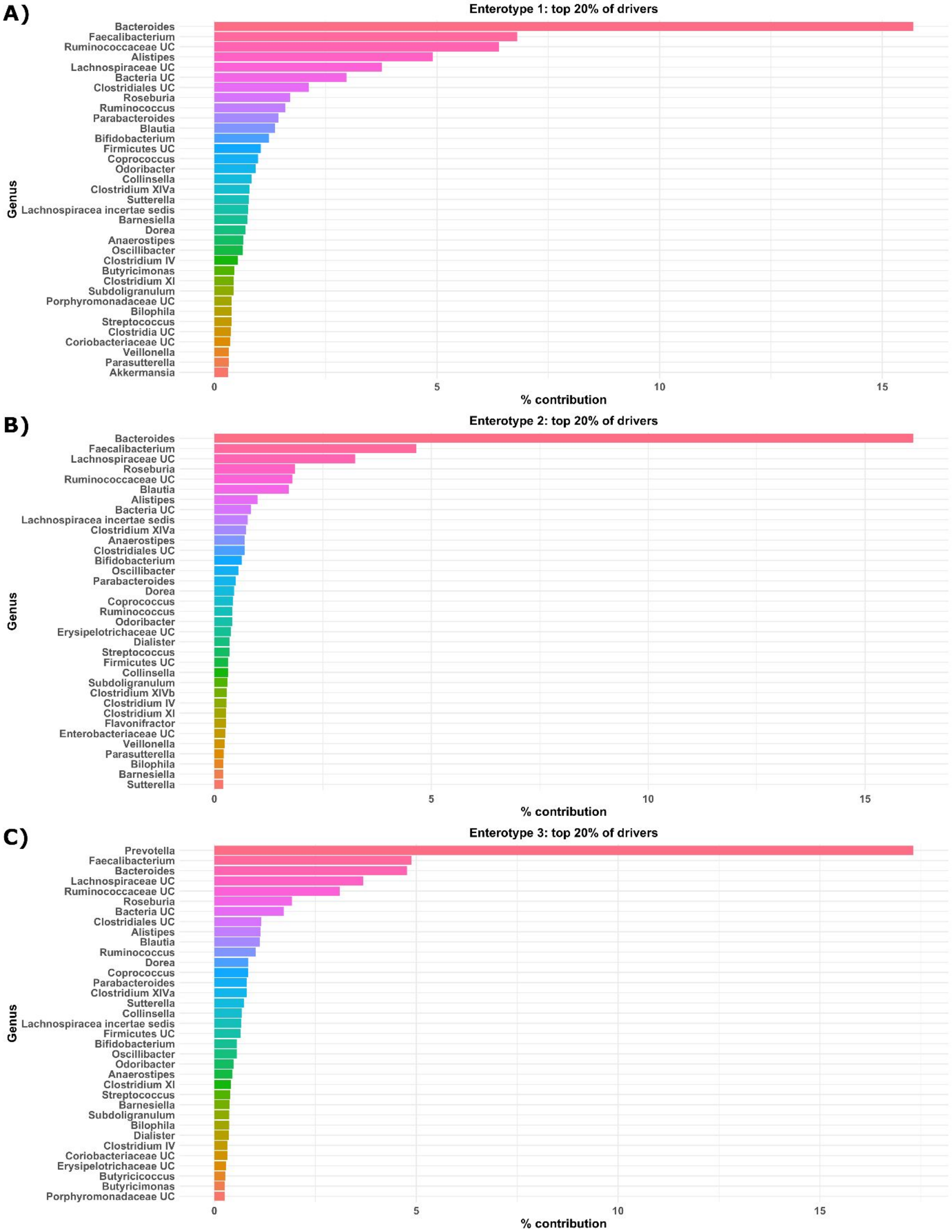

Supplementary Figure 2. Bar plots representing the top 20% of genera within each of the three identified microbial enterotypes. A) Enterotype 1, B) Enterotype 2, and C) Enterotype 3.

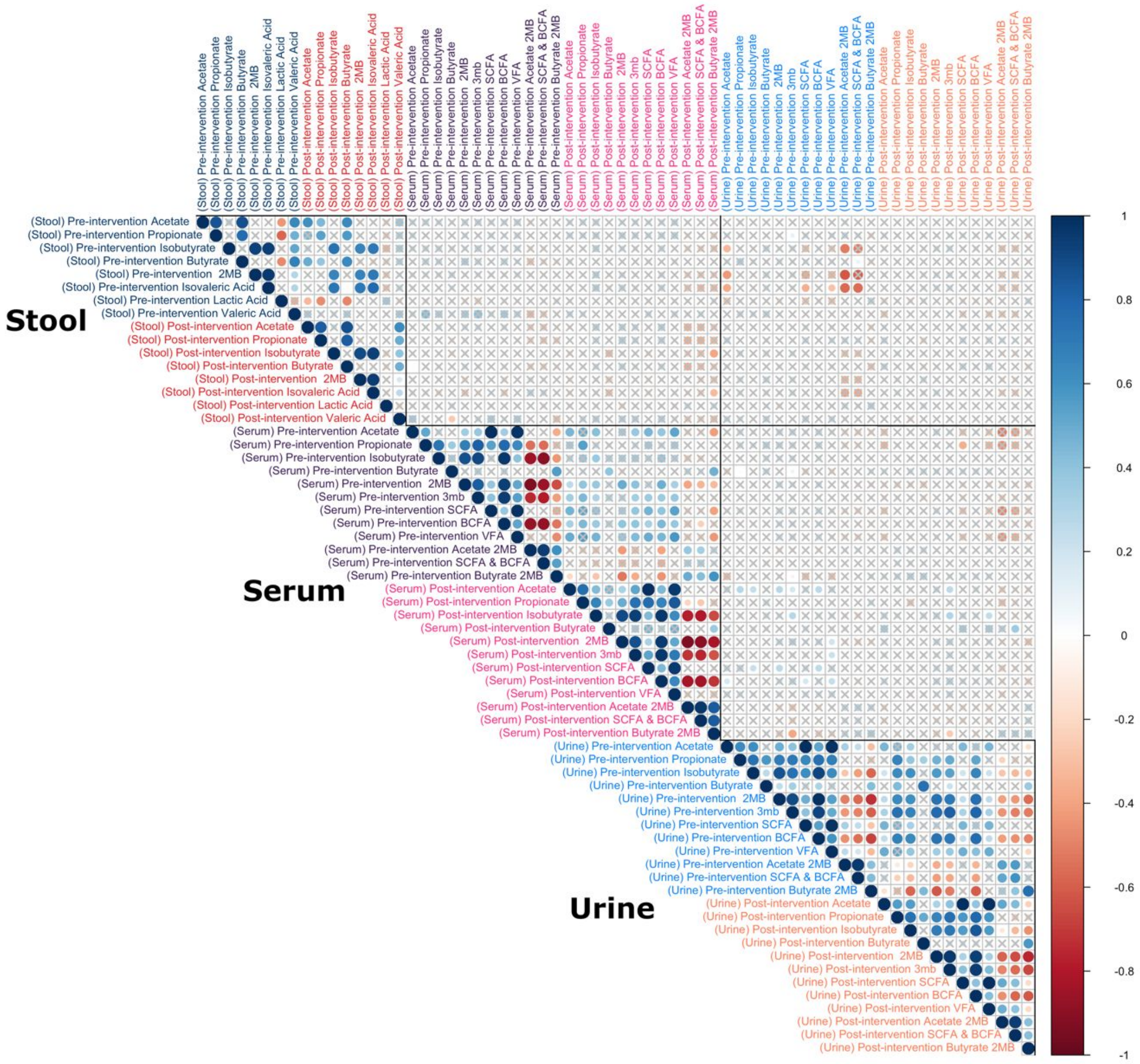

Supplementary Figure 3. Correlations between concentrations of pre- and post-intervention metabolites in plasma, stool, and urine. Colours of points represent Spearman's rho values, × within a point indicates a p value>0.05. Colours of labels represent pre-/post-intervention for each tissue type.

Abbreviations: 2MB, 2-methyl-1-butanol; 3MB, 3-methyl-1-butanol; SCFA, short-chain fatty acid; BCFA, branched-chain fatty acid.

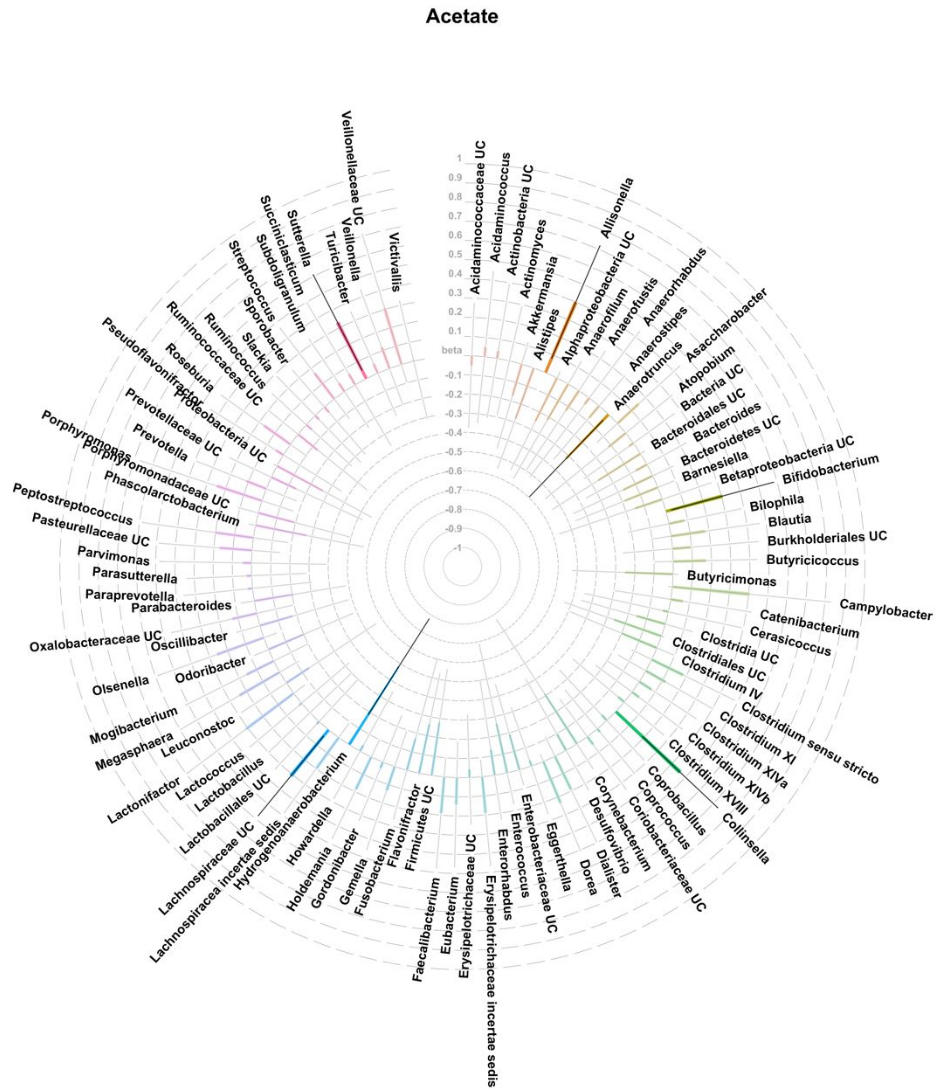

Supplementary Figure 4. Coefficients and 95% CIs for linear models between delta microbe abundances and delta Acetate concentrations.

Filled bars are nominally significant ($p < 0.05$) associations, semi-transparent bars are non-significant. It is important to note, no association passes multiple testing threshold (FDR <0.05).

Abbreviations: UC: uncultured.

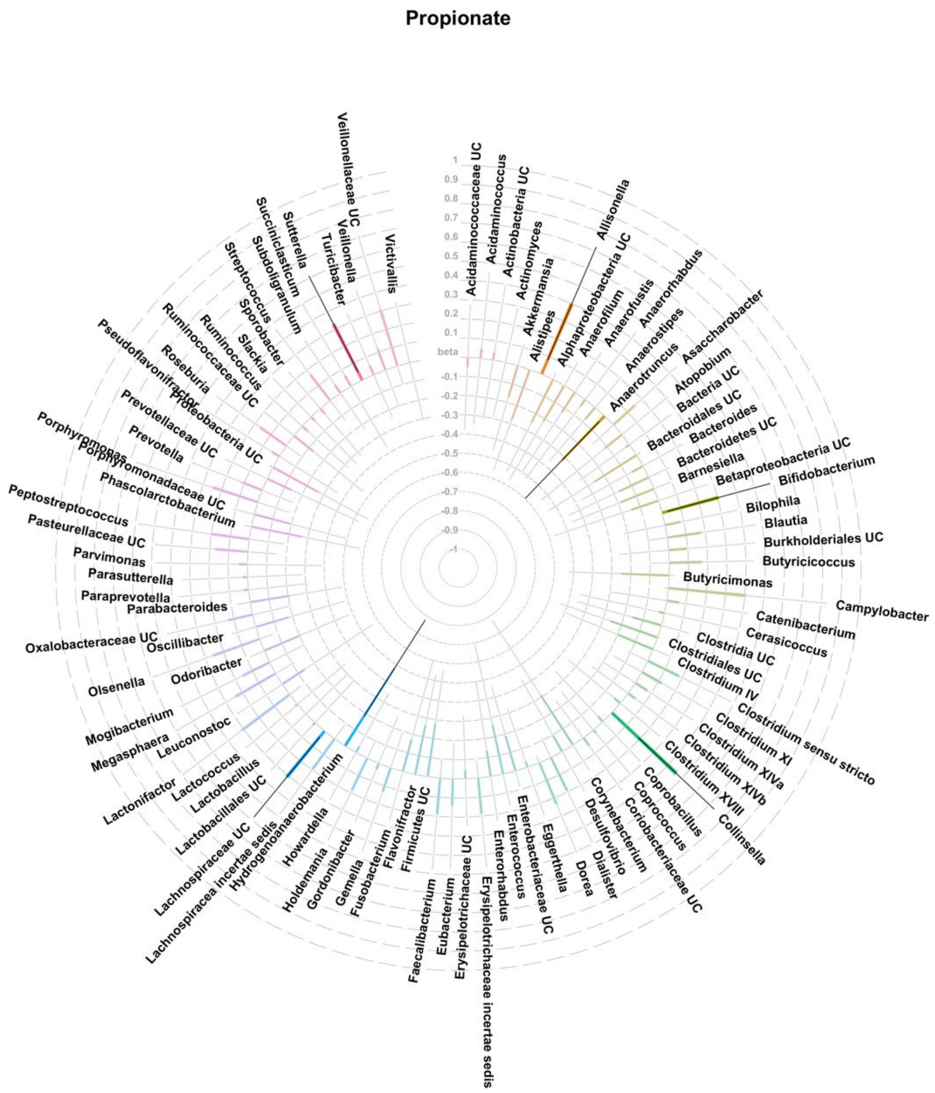

Supplementary Figure 5. Coefficients and 95% CIs for linear models between delta microbe abundances and delta Propionate concentrations.
Filled bars are nominally significant ($p < 0.05$) associations, semi-transparent bars are non-significant. It is important to note, no association passes multiple testing threshold (FDR <0.05).
Abbreviations: UC: uncultured.

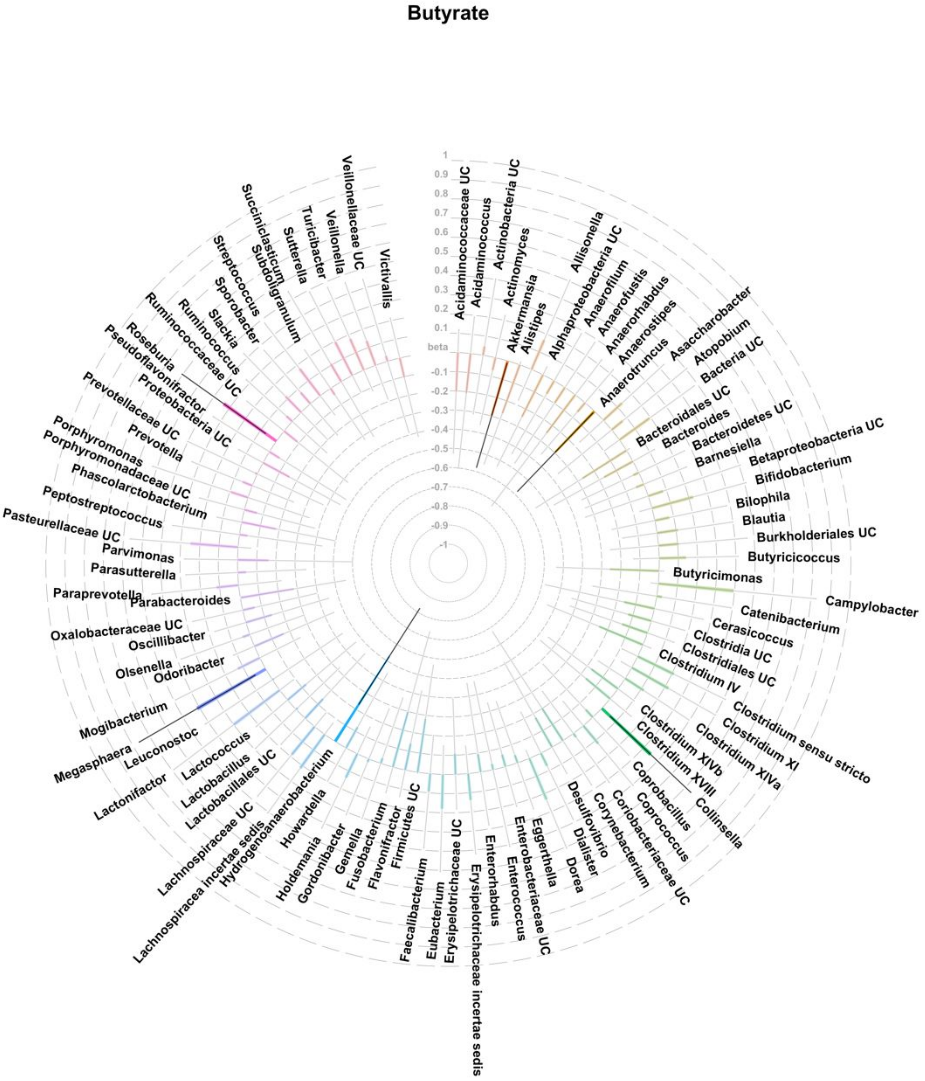

Supplementary Figure 6. Coefficients and 95% CIs for linear models between delta microbe abundances and delta Butyrate concentrations.
Filled bars are nominally significant ($p < 0.05$) associations, semi-transparent bars are non-significant. It is important to note, no association passes multiple testing threshold (FDR <0.05).
Abbreviations: UC: uncultured.
